# Small-molecule inhibitors of the PDZ domain of Dishevelled proteins interrupt Wnt signalling

**DOI:** 10.5194/mr-2-355-2021

**Published:** 2021-06-02

**Authors:** Nestor Kamdem, Yvette Roske, Dmytro Kovalskyy, Maxim O. Platonov, Oleksii Balinskyi, Annika Kreuchwig, Jörn Saupe, Liang Fang, Anne Diehl, Peter Schmieder, Gerd Krause, Jörg Rademann, Udo Heinemann, Walter Birchmeier, Hartmut Oschkinat

**Affiliations:** 1 Leibniz-Forschungsinstitut für Molekulare Pharmakologie, Robert-Rössle-Straße 10, 13125 Berlin, Germany; 2 Institut für Chemie und Biochemie, Freie Universität Berlin, Takustraße 3, 14195 Berlin, Germany; 3 Max Delbrück Center for Molecular Medicine, Robert-Rössle-Straße 10, 13125 Berlin, Germany; 4 Enamine Ltd., Chervonotkatska Street 78, Kyiv 02094, Ukraine; 5 Institut für Pharmazie, Freie Universität Berlin, Königin-Luise-Straße 2 + 4, 14195 Berlin, Germany; 6 ChemBio Ctr, Taras Shevchenko National University of Kyiv, 62 Volodymyrska, Kyiv 01033, Ukraine

## Abstract

Dishevelled (Dvl) proteins are important regulators of the Wnt signalling pathway, interacting through their PDZ domains with the Wnt receptor Frizzled. Blocking the Dvl PDZ–Frizzled interaction represents a potential approach for cancer treatment, which stimulated the identification of small-molecule inhibitors, among them the anti-inflammatory drug Sulindac and Ky-02327. Aiming to develop tighter binding compounds without side effects, we investigated structure–activity relationships of sulfonamides. X-ray crystallography showed high complementarity of anthranilic acid derivatives in the GLGF loop cavity and space for ligand growth towards the PDZ surface. Our best binding compound inhibits Wnt signalling in a dose-dependent manner as demonstrated by TOP-GFP assays (IC ${}_{50}∼50$ µM ) and Western blotting of β -catenin levels. Real-time PCR showed reduction in the expression of Wnt-specific genes. Our compound interacted with Dvl-1 PDZ (K ${}_{D}=2.4$ µM ) stronger than Ky-02327 and may be developed into a lead compound interfering with the Wnt pathway.

## 1 Introduction

Dishevelled (Dvl) proteins comprise 500 to 600 amino acids and contain three conserved domains: an N-terminal DIX (Dishevelled/Axin) domain (Schwarz-Romond et al., 2007; Madrzak et al., 2015), a central PDZ (PSD95/Dlg1/ZO-1) domain (Doyle et al., 1996; Ponting et al., 1997), and a C-terminal DEP (Dishevelled/Egl-10/Pleckstrin) domain (Wong et al., 2000; Wallingford and Raymond, 2005). Dvl transduces Wnt signals from the membrane receptor Frizzled to downstream components via the interaction between Dvl PDZ and Frizzled (Wong et al., 2003); thus, it has been proposed as a drug target (Klaus and Birchmeier, 2008; Holland et al., 2013; Polakis, 2012). Several studies identified internal peptides of the type KTXXXW as well as C-terminal peptides of the type ΩΦ GWF in which Ω is any aromatic amino acid (F, W, or Y) as Dvl PDZ targets (Lee et al., 2009a; Zhang et al., 2009). Three Dvl homologues, Dvl-1, Dvl-2, and Dvl-3, have been identified in humans. Sequence identity is 88 % between Dvl-3 PDZ and Dvl-1 PDZ and 96 % between Dvl-3 PDZ and Dvl-2 PDZ (Fig. S1 in the Supplement). Dvl proteins are found to be upregulated in breast, colon, prostate, mesothelium, and lung cancers (Weeraratna et al., 2002; Uematsu et al., 2003a, b; Bui et al., 1997; Mizutani et al., 2005).

**Figure 1:**
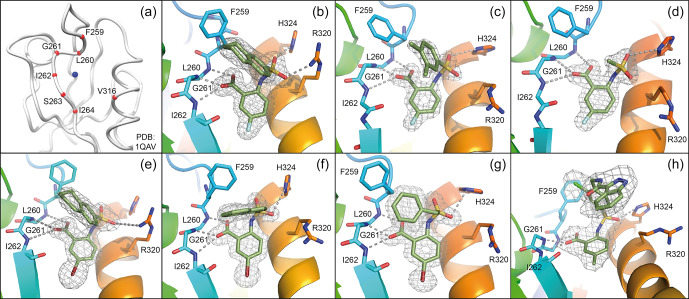
**(a)** Definition of PDZ binding site. The centre of the binding site (blue sphere) is defined as the geometric centre of C α atoms (red spheres) of seven residues (typed in red) defined by multiple sequence alignment. **(b–h)** Magnified views into crystal structures of various compounds bound to the Dvl-3 PDZ domain. The 2Fo-Fc electron density around the compounds is shown at 1 σ contour level, and the dotted lines indicate formed hydrogen bonds. In the bound compounds covalent bonds to carbon atoms are shown as green sticks. Important residues involved in compound binding are labelled and displayed in atom colours (carbons blue or dark yellow). Panels **(b–d)** show compound **3**, **5**, and **6**, respectively. All compounds in **(b–d)** contain fluorine (light blue) in para position to the amine. Panels **(e–g)** represent the bound compounds **7**, **11**, and **12**, respectively. All have bromine (dark red) in para position to the amine. Panel **(h)** shows compound **18** within the binding site. The accession codes of the structures **(b–h)** are 6ZBQ, 6ZBZ, 6ZC3, 6ZC4, 6ZC6, 6ZC7, and 6ZC8, respectively.

PDZ domains appear in 440 copies spread over more than 260 proteins of the human proteome (Ponting et al., 1997). They maintain relatively specific protein–protein interactions and are involved, for example, in signalling pathways, membrane trafficking, and in the formation of cell–cell junctions (Zhang and Wang, 2003; Fanning and Anderson, 1996; Kurakin et al., 2007). Hence, they are potentially attractive drug targets (Rimbault et al., 2019; Christensen et al., 2020). PDZ domains consist of about 90 amino acids which fold into two α -helices and six β -strands exposing a distinct peptide-binding groove (Doyle et al., 1996; Lee et al., 2017). The conserved carboxylate-binding loop (GLGF loop, FLGI in Dvl-2, and -3, Fig. 1) is essential for the formation of a hydrogen bonding network between the PDZ domain and PDZ-binding C-terminal peptide motifs, in most cases coordinating the C-terminal carboxylate group of the interaction partner. In the respective complexes, the C-terminal residue of the ligand is referred to as P ${}_{0}$ ; subsequent residues towards the N-terminus are termed P ${}_{-1}$ , P ${}_{-2}$ , and P ${}_{-3}$ , etc. Previous studies have revealed that P ${}_{0}$ and P ${}_{-2}$ are most critical for PDZ-ligand recognition (Songyang et al., 1997; Schultz et al., 1998).

PDZ domains are divided into at least three main classes on the basis of their amino acid preferences at these two sites: class I PDZ domains recognize the motif S/T-X- Φ -COOH ( Φ is a hydrophobic residue, and X is any amino acid), class II PDZ domains recognize the motif Φ -X- Φ -COOH, and class III PDZ domains recognize the motif X-X-COOH. However, some PDZ domains do not fall into any of these specific classes (Pawson, 2007; Sheng and Sala, 2001; Zhang and Wang, 2003). The Dvl PDZ domains, for example, recognize the internal sequence KTXXXW within the Frizzled peptide 525(GKTLQSWRRFYH)536 (K ${}_{D}∼10$ µM ) (Wong et al., 2003; Chandanamali et al., 2009).

Due to their occurrence in important proteins, PDZ domains received early attention as drug targets, which has been nicely summarized in Christensen et al. (2019). There are several examples of Dvl PDZ inhibitors of a peptide or peptidomimetic nature (e.g. Hammond et al., 2006; Haugaard-Kedstrom et al., 2021), including peptide conjugates (e.g. Qin et al., 2021; Hegedüs et al., 2021) and on an organic small-molecule basis. The latter approach is considered most beneficial in long-term medical treatments of conditions like cancer or neurological disorders. NSC668036 (Shan et al., 2005; Wang et al., 2015) is a peptide-mimic compound which interferes with Wnt signalling at the Dvl level. Based on a computational pharmacophore model of NCS668036, additional compounds were later reported (Shan et al., 2012). Known as the first non-peptide inhibitor, the 1H-indole-5-carboxylic acid derivative FJ9 (Fujii et al., 2007) showed therapeutic potential. Further examples including Sulindac (Lee et al., 2009b), 2-((3-(2-phenylacetyl)amino)benzoyl)amino)benzoic acid (3289-8625, also called CalBioChem(CBC)-322338) (Grandy et al., 2009; Hori et al., 2018), N-benzoyl-2-amino-benzoic acid analogues (Hori et al., 2018), phenoxyacetic acid analogue (Choi et al., 2016), and ethyl 5-hydroxy-1-(2-oxo-2-((2-(piperidin-1-yl)ethyl)amino)ethyl)-1H-indole-2-carboxylate (KY-02327) (Kim et al., 2016) have been reported, with the last one showing the highest in vitro affinity (8.3 µM ) of all. Despite the existence of the abovementioned inhibitors of Dvl PDZ, the development of tighter-binding non-peptidic small-compound modulators of the respective functions, binding with nanomolar affinity, is necessary and remains challenging. On this path, we explore optimal fits for the primary binding pocket by cycles of chemical synthesis and X-ray crystallography and explore further avenues for systematically growing ligands along the Dvl PDZ surface to provide structure–activity relationship (SAR) for the development of inhibitors in the low or medium nanomolar range. Nuclear magnetic resonance (NMR) spectroscopy was used to detect primary hits and for follow-up secondary screening. The ability of NMR to detect weak intermolecular interactions ( µM < K ${}_{D}<$ mM) makes it an ideal screening tool for identifying and characterizing weakly binding fragments, to be optimized subsequently by chemical modification in order to improve binding (Zartler and Shapiro, 2006; Shuker et al., 1996; Zartler et al., 2003). Besides NMR, the determination of X-ray crystal structures of selected complexes was fundamental for further design of new compound structures with improved binding. In the first round of screening, a library constructed after computational docking of candidates into the peptide binding site of the Dvl PDZ domains was investigated, followed by secondary screening utilizing a library of 120 compounds containing rhodanine or pyrrolidine-2,5-dione moieties.

## 2 Results and discussion

### 2.1 PDZ targeted library design

The PDZ targeted library was designed to cover all PDZ domains with available structure. For this, all X-ray- and NMR-derived PDZ structures were retrieved from the PDB, clustered, and six selected centroids were subjected to the virtual screening routine. The area considered is shown in Fig. 1a, with the blue sphere indicating the geometrical centre. The clustering of the PDZ domains was performed according to the shapes of their binding sites rather than backbone conformation. This approach accounts for the importance of surface complementarity of protein–small-molecule interactions and the critical contribution of van der Waals interactions to the binding free energy. On the other hand, PDZ domains have evolved to recognize a carboxyl group that is mostly derived from the C-terminus of natively binding proteins. Finally, the fact that PDZ can recognize internal motifs (Hillier et al., 1999), including KTXXXW of Frizzled-7 recognized by Dvl PDZ (Wong et al., 2003; Chandanamali et al., 2009), raises the question of what are key binding contributions with PDZ domains: negative charge, hydrogen bonding, or shape complementarity (Harris et al., 2003). For this reason, tangible compounds were preselected to have extensive hydrophobic contacts as well as chemical groups that mimic the carboxylic group.

Virtual screening was performed with QXP (McMartin and Bohacek, 1997), and the generated complexes were sequentially filtered with a self-designed MultiFilter algorithm. From the resulting 1119 compounds a randomly selected set of 250 compounds was subjected to NMR validation.

### 2.2 NMR screening and development of compounds

The results of virtual screening were checked experimentally by comparing 2D ${}^{1}H{-}^{15}N$ HSQC (heteronuclear single quantum correlation) spectra of Dvl-3 PDZ in the absence and presence of the compound to elucidate ligand-induced changes of chemical shifts. Chemical shift perturbation differences ( Δ CSP, representing the average of the three strongest shifting cross peaks according to Eq. 1) were evaluated in cases where the residues responding strongest are inside the area defined by Fig. 1a. The responses were classified into (i) inactive compounds ( Δ CSP <0.02 ); (ii) very weak interactions ( 0.02≤ΔCSP≤0.05 ); (iii) weak interactions ( 0.05<ΔCSP≤0.1 ); (iv) intermediate interactions ( 0.1<ΔCSP≤0.2 ); (v) strong interactions ( 0.2<ΔCSP≤0.5 ), and (vi) very strong interactions ( ΔCSP>0.5 ). In most cases, the signals of residues S263, V287, and R320 (Fig. 1a) within the conserved binding site were most strongly perturbed (Fig. S2). With the Δ CSP of 0.12 ppm, the isoleucine-derived compound **1** ((2,3-dihydrobenzo[b][1,4]dioxin-6-yl)sulfonyl)-L-isoleucine containing a sulfonamide moiety was detected initially as one of the best “hits” according to chemical shift changes. The sulfonamide is a well-known moiety in drug discovery (Mathvink et al., 1999; Wu et al., 1999; Sleight et al., 1998; O'Brien et al., 2000; Tellew et al., 2003).


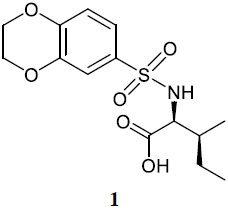


Upon NMR titration experiments for compound **1** (Fig. S2) with Dvl-3 PDZ, the largest chemical shift perturbations were observed for S263 in strand β B and R320 in helix α B of Dvl-3 PDZ, confirming the conserved binding site.

**Scheme 1:**
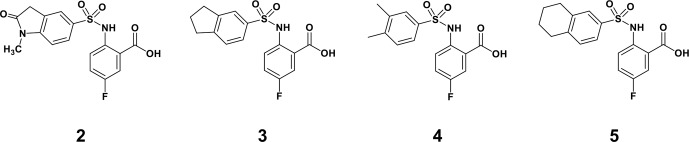
Compounds **2**, **3**, **4**, and **5**.

**Scheme 2:**
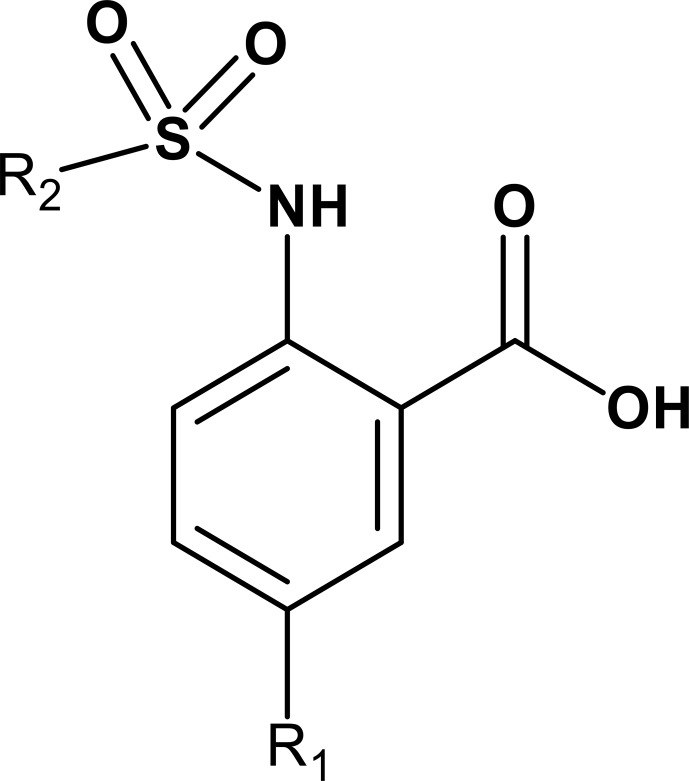
Basic fragment for further synthesis.

By comparing the binding of several sulfonamide compounds in a secondary screening event and making use of our in-house library, four new compounds (**2**, **3**, **4**, **5**) that induced chemical shift perturbations larger than 0.2 ppm were found (for binding constants see Table 1) and considered further as reasonably strong binders. The similarity of the structures led us to define Scheme 2 as a scaffold for further refinements. Sulfonamides were considered more drug-like and hence followed up at higher priority than other hits. We realized that our four new compounds had different moieties at R ${}_{2}$ in combination with a small R ${}_{1}$ (fluorine). A decrease of binding was observed with decreasing size of R ${}_{2}$ .

In order to assess the importance of the aryl group at R ${}_{2}$ for complex formation, it was replaced by a methyl group as substituent to yield compound **6**, which showed a drastic decrease of binding (Table 1). Compounds **3**, **4**, and **5** did not distinguish between the Dvl-3 PDZ and Dvl-1 PDZ. In order to obtain detailed insight into the binding mode of these compounds, crystal structures of Dvl-3 PDZ in complex with compounds **3**, **5**, and **6** were determined (Fig. 1). For compound **3**, the crystal structure revealed two complexes within the crystallographic asymmetric unit (AU) at 1.43 Å resolution. Both show the anthranilic acid with the attached fluorine pointing into the hydrophobic binding pocket (Figs. 1b and S3a), while the carboxyl group forms a hydrogen-bond network with amide residues of the carboxylate binding loop, in particular strand β B (Fig. 1b) and specifically with residues I262, G261, and L260. The two sulfonamide oxygen atoms form hydrogen bonds with R320 and H324 (weak) of helix α B for only one complex in the AU. The aromatic aryl group (tetrahydronaphtalene) attached to the sulfonamide is involved in hydrophobic interactions with F259 were (Fig. S3b). The 1.6 Å complex structure with compound **5** (four molecules per AU) exhibits a comparable binding mode as found for compound **3** with a hydrogen-bond network involving the carboxyl group and the amides of I262, G261, L260, and of the sulfonamide to H324 (Fig. 1c). No hydrogen bond was observed to R320 in all four molecules of the AU, but small variations of the aryl moiety relative to F259 (Fig. S3c). The crystals of the complex with **6** show two molecules in the AU (Fig. 1d). The sulfonamide is bound by H324 in both complexes (Fig. S3d). However, compound **6** bound only in the millimolar (mM) range as compared to **3** and **5**, which obviously results from the missing aromatic rings.

**Table 1:** Binding constants K ${}_{D}$ ( µM ) of Dvl-3 PDZ and Dvl-1 PDZ for compounds **3**–**21** derived by ITC or NMR if not specified. The K ${}_{D}$ values determined by NMR are reported as means ± standard deviations of measurements evaluating shifts of cross peaks of at least six residues influenced upon binding of the ligand. The K ${}_{D}$ values ( $1/K_{A}$ ) determined by ITC were obtained as fits to a one-site binding model ( n in the range of 0.95–1.2) with K ${}_{D}$ errors obtained by $\Delta {K_{A}/K_{A}}^{2}$ .

	ID	R ${}_{1}$	R ${}_{2}$	(K ${}_{D}$ , µM )	(K ${}_{D}$ , µM )
				Dvl-3 PDZ	Dvl-1 PDZ
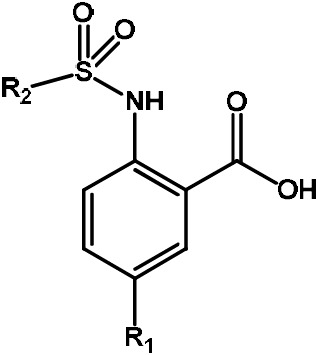	3	F	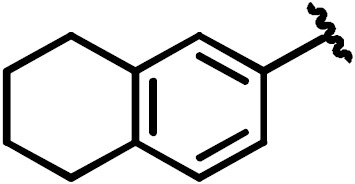	$80.6\pm 6.1^{\mathrm{NMR}}$	$112.7\pm 25.9^{\mathrm{NMR}}$
	4	F	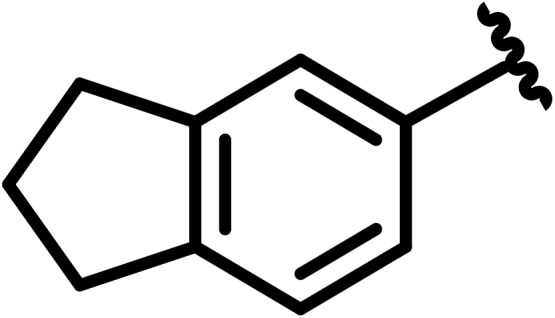	$83.9\pm 7.8^{\mathrm{NMR}}$	$114.4\pm 9.8^{\mathrm{NMR}}$
	5	F	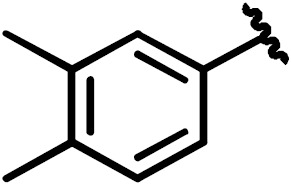	$140.6\pm 14.1^{\mathrm{NMR}}$	$160.1\pm 14.6^{\mathrm{NMR}}$
	6	F	CH ${}_{3}$	$>1000^{\mathrm{ITC}}$	–
	7	Br	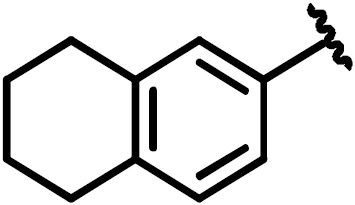	$20.6\pm 2.4^{\mathrm{NMR}}$	$18.2\pm 2.4^{\mathrm{NMR}}$
	8	CF ${}_{3}$	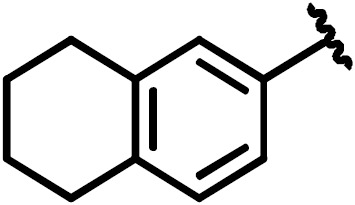	$17.4\pm 0.5^{\mathrm{ITC}}$	$24.5\pm 1.5^{\mathrm{ITC}}$
	9	Cl	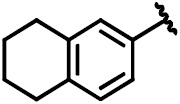	$41.1\pm 3.1^{\mathrm{NMR}}$	$45.6\pm 4.5^{\mathrm{NMR}}$
	10	CH ${}_{3}$	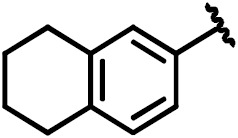	$62.5\pm 4.7^{\mathrm{NMR}}$	$60.5\pm 5.3^{\mathrm{NMR}}$
	11	Br	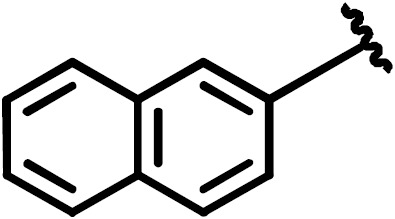	13.8 ${}^{\mathrm{ITC}}$	119.9 ${}^{\mathrm{ITC}}$
	12	Br	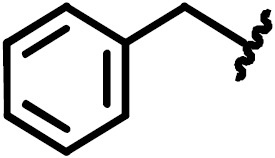	58.5 ${}^{\mathrm{ITC}}$	nd
	13	Br	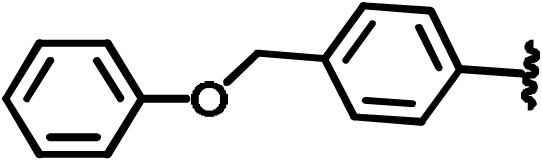	$7.2^{\mathrm{ITC}}$	213.2 ${}^{\mathrm{ITC}}$
	14	Br	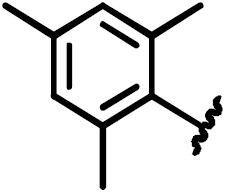	$58.1\pm 2.1^{\mathrm{ITC}}$	nd
	15	CF ${}_{3}$	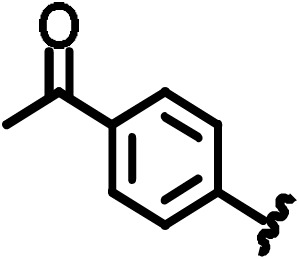	$52.9\pm 1.7^{\mathrm{ITC}}$	nd
	16	CF ${}_{3}$	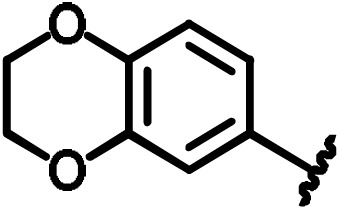	$59.1\pm 1.5^{\mathrm{ITC}}$	nd
	17	CF ${}_{3}$	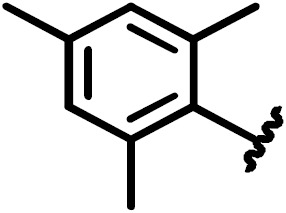	49.5 ${}^{\mathrm{ITC}}$	nd
	18	CH ${}_{3}$	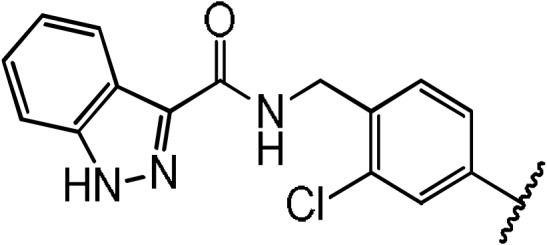	$9.4\pm 0.6^{\mathrm{ITC}}$	$2.4\pm 0.2^{\mathrm{ITC}}$
	19	CH ${}_{3}$	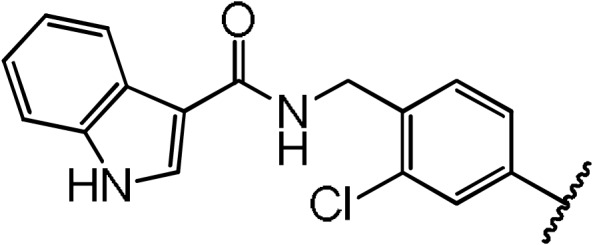	$21.8\pm 1.7^{\mathrm{ITC}}$	$8.0\pm 0.5^{\mathrm{ITC}}$
	20	CH ${}_{3}$	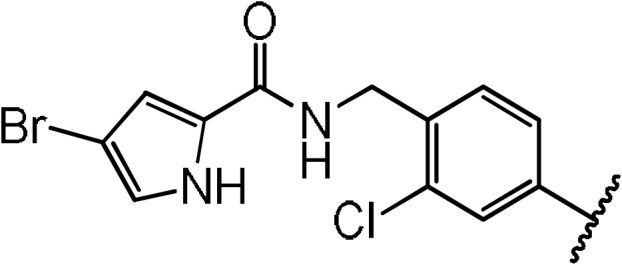	$9.8\pm 0.3^{\mathrm{ITC}}$	$4.7\pm 0.3^{\mathrm{ITC}}$
	21	CH ${}_{3}$	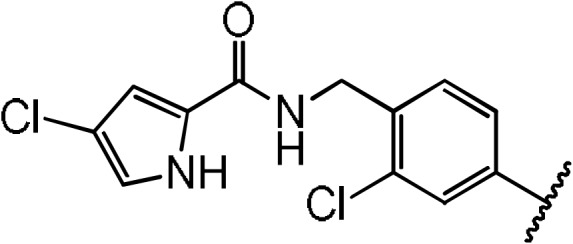	$12.5\pm 0.5^{\mathrm{ITC}}$	$4.7\pm 0.2^{\mathrm{ITC}}$

nd: not determined.

To further explore the importance of the fluorine site inside the hydrophobic pocket, substitutions by bromine, chlorine, methyl, and trifluoromethyl were chosen. In fact, the methyl group has a similar van der Waals (vdW) radius as the CF ${}_{3}$ group. Iodine was not considered a good candidate since it increases molecular weight substantially, and the compounds may be chemically less stable, in particular in biological assays. Taking into account that compound **6** did not bind because of the missing aromatic ring at the R ${}_{2}$ position, our overall strategy was to increase the aromatic ring at R ${}_{2}$ while finding a good fit for R ${}_{1}$ , keeping an eye on the molecular weight to enable further compound modifications that fulfil key properties as defined by Lipinski (2000) and Lipinski et al. (1997). Our preference to continue exploration at the R ${}_{1}$ position of the aromatic ring in Scheme 1 was inspired by the absence of hits with other substitutions in the secondary screening event and the initial X-ray structures that showed a hydrophobic pocket available for substituents in this position, while other sites at the aromatic ring would include steric hindrance. Therefore, compounds **7**–**17** were obtained and were classified in three different groups to derive structure–activity relationships (SARs). The compounds **7**–**10** in group 1 contain different R ${}_{1}$ (Br, CF ${}_{3}$ , Cl, CH ${}_{3}$ ) but the same moiety (tetrahydronaphthalene) at R ${}_{2}$ . As expected, binding could be further improved by displacement of the fluorine with elements exhibiting larger vdW radii. Indeed, the K ${}_{D}$ decreased stepwise, and the best fit was observed for compound **8** containing a trifluoromethyl group (K ${}_{D}=17.4$ µM for Dvl-3 PDZ and 24.5 µM for Dvl-1 PDZ). The different substituents at the R ${}_{1}$ position contribute to an increased binding affinity in the following order: $F<\mathrm{Cl}<\mathrm{Br}<{\mathrm{CF}}_{3}$ (compound **3** < **9** < **7** < **8**, respectively). Compound **10** with a methyl group at the R ${}_{1}$ position showed only marginally improved binding, although the methyl group has a similar vdW radius as the CF ${}_{3}$ group of compound **8**. The difference in binding results most likely from their different hydrophobicity.

The 1.85 Å crystal structure of the Dvl-3 PDZ domain with compound **7** (K ${}_{D}=20.6$ µM for Dvl-3 PDZ and 18.2 µM for Dvl-1 PDZ) showed an identical hydrogen-bond network involving the amide groups of residues I262, G261, and L260 of the carboxyl binding loop as seen for all other complex structures reported here (Fig. 1e). Only one hydrogen bond between the sulfonamide and R320 was found in addition for one of the two Dvl-3 PDZ molecules per AU. H324 of Dvl-3 PDZ was not addressed by the sulfonamide as seen previously. The bromine at position R ${}_{1}$ points into the hydrophobic pocket, which is similar as the fluorine in the complex structure with compound **3**. The two complexes in the AU show significant variations in the positions of the tetrahydronaphtalene rings as well as for the side chain of F259 and R320 (Fig. S3e).

Following the analysis of the complex involving compound **7**, the binding characteristics of the group-2 compounds (**11**–**14**) were investigated. They contain bromine as R ${}_{1}$ and different substituents at the R ${}_{2}$ position to assess the importance of π – π stacking interactions involving F259. K ${}_{D}$ values of 7.2 µM for compound **13** and 13.8 µM for compound **11** were found with respect to the interaction with Dvl-3 PDZ. Crystal structures of Dvl-3 PDZ in complex with compound **11** (1.58 Å resolution, 1 molecule per AU) and **12** (1.48 Å, 2 molecules per AU) revealed very similar binding as observed in the crystal structures with compounds **3** and **7**. The aromatic rings at R ${}_{2}$ show hydrophobic interactions to F259 but not a classical π – π stacking as expected. Nevertheless, the tighter binding of compound **11** could be explained by the larger aromatic substituent at the R ${}_{2}$ position compared to compound **12**. Both complex structures show also non-specifically bound ligands in crystal contacts (Fig. S3h, Tables S2 and S3 in the Supplement). The additional ligand molecules in both complex structures can be explained as a crystallographic artefact, which is verified with ITC experiments that indicate 1:1 stoichiometries in both cases (Fig. S5). With respect to the selectivity of the tested compounds, we observed a 6- to 30-fold stronger binding of compounds **7**, **9**, **11**, and **13** to Dvl-3 PDZ as compared to Dvl-1 PDZ. These differences are related to the different sequences at the end of α B. Most importantly, H324 is replaced by a serine residue in the Dvl-1 PDZ domain.

The group-3 compounds (**15**–**17**) contain a trifluoromethyl at position R ${}_{1}$ and were tested to investigate a cooperative role of this moiety with various substituents at position R ${}_{2}$ . All compounds bind weaker to Dvl-1 and Dvl-3 than compound **8** which contains tetrahydronaphthalene at the R ${}_{1}$ position, revealing its important role in the interaction.

### 2.3 Further modifications towards higher affinity and reduced toxicity

Possible cytotoxic effects of compounds **3**, **7**, **8**, **9**, and **10** were evaluated in cell viability assays using HEK293 cells (Fig. S4). These compounds were selected due to different substituents at the R ${}_{1}$ , including halogens. Cell viability was measured 24 h after treatment with the individual compounds, and half maximal inhibitory concentrations (EC ${}_{50}$ ) were calculated for each compound. The compounds exhibited EC ${}_{50}$ values in the range of 61–131 µM (Fig. S4a). Compounds **3** and **10** that contained fluorine or methyl group substituents at R ${}_{2}$ , respectively, were the least toxic, while compound **7**, containing bromine, was the most toxic. The results from crystallography, modelling studies, and of the cell proliferation assays led us to further investigate compounds **18**–**21** that contain a methyl group at the R ${}_{1}$ position and different substituents as R ${}_{2}$ . In this way, we aimed to develop both potent and less-toxic cell-permeable inhibitors. All compounds showed strong interactions as indicated by chemical shift perturbation values between 0.30 to 0.34 ppm (Table S1). The binding constants were evaluated by ITC whereby compound **18** (K ${}_{D}=9.4$ µM for Dvl-3 PDZ and 2.4 µM for Dvl-1 PDZ) appeared to be most potent. Compound **18** contains a pyrazole ring which is considered an important biologically active heterocyclic moiety (Lv et al., 2010). Compounds **20** (K ${}_{D}=9.8$ µM for Dvl-3 PDZ and 4.7 µM for Dvl-1 PDZ) and **21** (K ${}_{D}=12.5$ µM for Dvl-3 PDZ and 4.7 µM for Dvl-1 PDZ) contain pyrrole rings. Their binding constants almost have the same value despite the different substituents (bromine or chlorine) at the pyrrole rings. The binding of compounds **18**–**21** to both Dvl PDZ domains is mainly enthalpy-driven as indicated in Table 2, with a slightly stronger effect for Dvl-1 PDZ than for Dvl-3 PDZ. To our surprise, the crystal structure of Dvl-3 PDZ in complex with compound **18** shows the pyrazole substituent in the R ${}_{2}$ position orientated away from the binding pocket. Instead, a π – π stacking interaction with F259 was observed (Fig. S3i). Cytotoxicity of **18**–**21** was determined via MTT assays (Mosmann, 1983) that displayed viability up to concentrations above 150 µM (Fig. S4b).

**Table 2:** Isothermal titration calorimetric data for the reaction between Dvl-3 PDZ; Dvl-1 PDZ; and our compounds **18**, **19**, **20**, and **21**. Compounds NPL-1011 (Hori et al., 2018), Sulindac (Lee et al., 2009), CBC-322338/3289-8625 (Grandy et al., 2009; Hori et al., 2018), and NSC668036 (Shan et al., 2005) are also given; for more thermodynamic parameters, see Fig. S7. For Ky-02327, the value from literature is included.

Compound	Dvl-3 PDZ				Dvl-1 PDZ			
	K ${}_{D}$	Δ H	T Δ S	Δ G	K ${}_{D}$	Δ H	T Δ S	Δ G
	( µM )	(kcal mol ${}^{-1}$ )			( µM )	(kcal mol ${}^{-1}$ )		
**18**	9.4±0.6	-8.0	-1.2	-6.7	2.4±0.2	-12.2	-4.7	-7.5
**19**	21.0±1.7	-5.9	0.4	-5.5	8.0±0.5	-7.3	-0.3	-7.0
**20**	9.8±0.3	-10.4	-3.6	-6.8	4.7±0.3	-9.4	-2.2	-7.2
**21**	12.5±0.5	-5.9	0.7	-6.8	4.7±0.2	-8.5	-1.5	-7.0
NPL-1011	79.7±53.3							
Sulindac	8.3±2.5							
CBC-322338/3289-8625	>400 µM							
NSC668036	>400 µM							
Ky-02327					8.3±0.8			
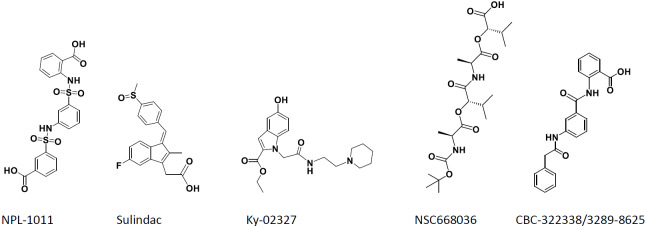								

### 2.4 Comparison to reported Dvl PDZ-binding molecule subsection

Our compounds bind to Dvl-3 with a K ${}_{D}$ better than 10 µM (and slightly tighter to Dvl-1, see Table 2) with **18** showing a K ${}_{D}$ of 2.4 µM and chemical shift changes indicating binding to the canonical binding site (Fig. 1a). For comparison, four compounds of those shown in Fig. S6 were assayed by ITC (Fig. S7), regarding their affinity to Dvl-3 PDZ. Ky-02327 was already determined to bind with a K ${}_{D}$ of 8.3±0.8 µM (Kim et al., 2016) to Dvl-1 PDZ. Our first interest was oriented towards sulfonamides. Hori et al. (2018) have recently reported 3-( { 3-[(2-carboxyphenyl)sulfamoyl]phenyl } sulfamoyl)benzoic acid (NPL-1011) binding to Dvl-1 PDZ via the detection of chemical shift changes and further sulfonamide compounds that showed smaller effects, indicating weaker binding. We examined the binding constant of NPL-1011 which possesses two sulfonamide moieties by ITC and found a value of 79.7±53.3 µM (see Table 2). For further comparisons, we assayed also CBC-322338/3289-8625, Sulindac, and NSC668036 by ITC. Surprisingly, CBC-322338/3289-8625 showed very low affinity, with a K ${}_{D}$ above 400 µM in our ITC assay, in line with the value found by Hori et al. (2018) ( 954±403 µM ). We also applied an NMR shift assay (Fig. S8), yielding a Δ CSP around 0.1. Based on NMR and ITC studies, the binding affinity of CBC-322338/3289-8625 to Dvl-3 seems to be less than 50 µM (comparing the CSPs from the NMR assay with those of our other compounds listed in Table S1 and the respective binding constants in Table 1, considering also the weak heat development in our ITC assay), which was larger than the originally reported value ( 10.6±1.7 ) (Grandy et al., 2009) that was obtained with a different method. Concerning non-sulfonamide compounds, a K ${}_{D}$ of 8.3±2.5 µM was detected for Sulindac, while NSC668036 (Shan et al., 2005) did not show high-affinity binding. These results are largely in agreement with literature. In all cases, compounds were tested for purity after K ${}_{D}$ measurements (see Fig. S9a–d).

### 2.5 Selectivity testing using a set of selected PDZ domains

Compounds **18**, **20**, and **21** were tested towards other PDZ domains for selectivity. The set included PSD95-PDZ 2 and 3, Shank-3, α -syntrophin, and AF-6 PDZ. According to the determined chemical shift perturbations (Table S4), our compounds show no or very weak interactions with the selected PDZ domains ( 0.05<ΔCSP≤0.1 ppm). These findings led to the conclusion that our compounds show considerable selectivity towards Dvl PDZ domains. This selectivity might be due to a unique feature of Dvl PDZ where R320 (Dvl-3 PDZ) or R322 (Dvl-1 PDZ) is crucial for interactions, explaining selectivity with respect to other PDZ domains. In addition, the large hydrophobic cavity for the side chain of the C-terminal residue of the interacting peptide is occupied by a large moiety in the case of compounds **18**, **20**, and **21** which might not be accommodated in most other PDZ domains.

### 2.6 Dvl inhibitors antagonize canonical Wnt signalling and Wnt-related properties of cancer cells

**Figure 2:**
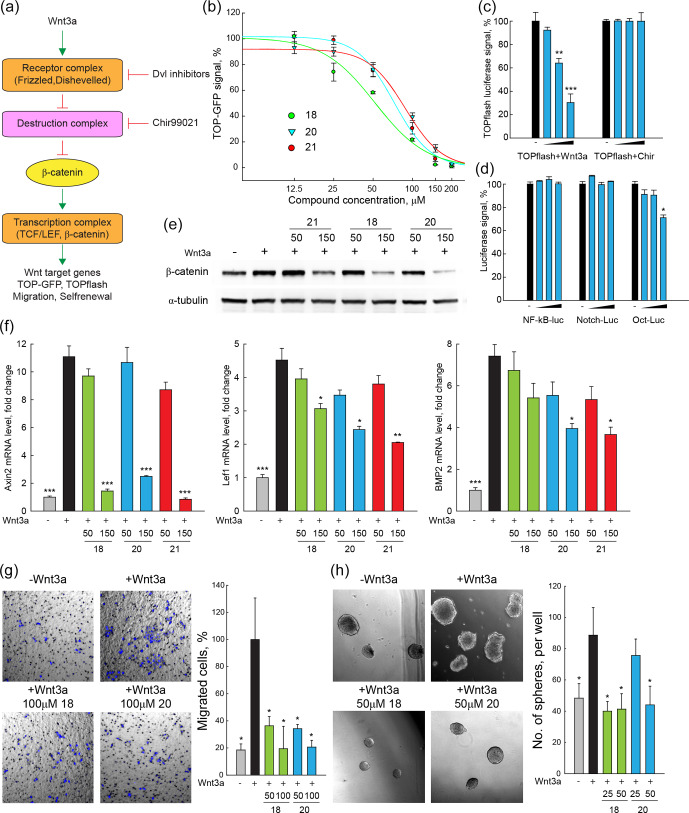
DVL inhibitors antagonize Wnt signalling and Wnt-related properties of cancer cells induced by Wnt3a. **(a)** Scheme of Wnt signalling pathway. Important components of the Wnt signalling pathway are schematically presented. Wnt3a treatment increases the transcription of Wnt targets, enhances signals of TOP-GFP and TOPflash assays, and promotes Wnt-related biological properties of cancer cells. **(b)** TOP-GFP reporter assays were performed with HEK293 reporter cell line. Compounds **18**, **20**, and **21** inhibited Wnt3a-induced Wnt activation in a dose-dependent manner with IC ${}_{50}$ of 50–75 µM . **(c, d)** TOPflash assays stimulated with Chir99021 and reporter assays of other pathways were used to evaluate the specificity of compound **20**. Compound **20** specifically inhibited Wnt3a-induced Wnt activation and had no or a mild effect on Chir99021-induced Wnt activation and other signalling pathways including NF– κ B, Notch, and Oct4. **(e)** β -Catenin protein levels were detected with Western blotting in HeLa cells. Compounds **18**, **20**, and **21** (150 µM ) inhibited accumulation of β -catenin in HeLa cells treated with Wnt3a. **(f)** The mRNA levels of Wnt target genes (Axin2, LEF1, and Bmp2) in HeLa cells were measured with quantitative real-time PCR. Compounds **18**, **20**, and **21** (150 µM ) reduced the transcription of Wnt target genes that are enhanced by Wnt3a treatment in HeLa cells. **(g)** Cell migration of SW480 cells after Wnt3a treatment was assessed by trans-well assays. Compounds **18** and **20** (50–100 µM ) reduced the migration of SW480 cells enhanced by Wnt3a. **(h)** SW480 cells were cultured in serum-free non-adherent conditions to evaluate the self-renewal property enhanced by Wnt3a treatment. Compounds **18** and **20** (25–50 µM ) reduced sphere formation of SW480 cells that was enhanced by Wnt3a treatment. For all tests, three independent biological replicas were performed and error bars represent standard deviations. P values were calculated from t test. ${}^{*}$ : P<0.05 ; ${}^{**}$ : P<0.01 ; ${}^{***}$ : P<0.001 .

Taking advantage of a lentivirus that encodes GFP in a β -catenin/TCF-dependent fashion (TOP-GFP, SABiosciences), a stable HEK293 reporter cell line was established to evaluate the inhibitory effect of compounds **18**, **20**, and **21** on canonical Wnt signalling activity. TOP-GFP expression in this cell line was induced by the ligand Wnt3a, which directly activates the Frizzled–Dishevelled complex and protects β -catenin from degradation by the destruction complex (Fig. 2a). Remarkably, all three compounds inhibited Wnt signalling induced by Wnt3a in a dose-dependent manner (Fig. 2b), yielding IC ${}_{50}$ values between 50–80 µM .

To further evaluate the specificity of our Dvl inhibitors, the conventional TOPflash (Molenaar et al., 1996) and other luciferase reporter assays were performed. In HeLa cells, 20 inhibited TOP-luciferase signals stimulated by Wnt3a but not by CHIR99021 (Sineva and Pospelov, 2010), a compound that activates Wnt signalling downstream of Dvl (Fig. 2a, c). Compound **20** had no significant inhibitory effects in reporter assays that measure the activity of other signalling systems, e.g. NF– κ B–luciferase stimulated by recombinant TNF α , Notch–luciferase stimulated by the overexpression of the Notch intracellular domain, or the Oct–luciferase assay that is stimulated by overexpression of Oct4 (SABiosciences, Fig. 2d). These results strongly indicate that 20 is specific for canonical Wnt signalling at the upstream level.

Increased β -catenin protein level is a hallmark of active Wnt signalling (Kishida et al., 1999). Once β -catenin is accumulated in the cytoplasm, it can translocate into the nucleus and activate the transcription of Wnt target genes by interacting with transcription factors of the TCF/LEF family (Fig. 2a) (Behrens et al., 1996). In HeLa cells, all three Dvl inhibitors blocked the increase of production of β -catenin by Wnt3a in a dose-dependent manner, as seen by Western blotting (Fig. 2e). Increased mRNA levels of the Wnt target genes Axin2, LEF1, and Bmp2 (Riese et al., 1997; Jho et al., 2002; Lewis et al., 2010) were induced by Wnt3a treatment, as measured by qRT-PCR, and these increases were reduced by compounds **18**, **20**, and **21** (Fig. 2f). These results demonstrate that compounds **18**, **20**, and **21** inhibit Wnt signalling as indicated by reduced accumulation of β -catenin and low expression of typical Wnt target genes.

Canonical Wnt signalling contributes to cancer progression by inducing high motility and invasion of cancer cells while retaining the self-renewal property of cancer-initiating cells (Fritzmann et al., 2009; Sack et al., 2011; Vermeulen et al., 2010; Malanchi et al., 2008). In particular, cancer-initiating cells are propagated and enriched in non-adherent sphere culture, demonstrating the self-renewal capacity of the stem cells (Kanwar et al., 2010; Fan et al., 2011). To investigate the potential value of the Dvl inhibitors for interfering with these Wnt-related properties of cancer cells, the sub-line SW480WL was derived from the SW480 colon cancer cell line, which exhibits a low level of endogenous Wnt activity (Fang et al., 2012). The cell migration and self-renewal properties of SW480WL cells were enhanced by Wnt3a treatment, as revealed by trans-well and sphere formation assays (Fig. 2g, h). Compounds **18** and **20** prevented increased cell migration and sphere formation. These results indicate that our Dvl inhibitors may be developed into lead compounds that interfere with Wnt signalling.

## 3 Experimental section

### 3.1 Clustering binding sites and selection of representative PDZ domains

Three-dimensional structures of PDZ domains were retrieved from the PDB (Berman et al., 2000). At the time of the study, from a total of 266 PDB files 126 were NMR solution structures and 140 were derived from X-ray diffraction studies. The structures belong to 163 PDZ domains of 117 different proteins from 11 organisms. Files which contain more than one 3D conformation for a domain (up to 50 for NMR-derived data) were split into separate structures and were considered independently. The total number of unique 3D structures was 2708.

Amino acid sequences of PDZ domains were aligned using Clustal Omega software (Sievers et al., 2011). Based on the alignment, for each structure, residues which form the binding site (strand β B and helix α B) were determined (Fig. S8). The centre of the binding site was defined as a geometric centre of C α atoms of seven residues (six residues from the β B strand and the second residue from the α B helix). Such bias towards the β B strand was made to cover sites occupied by residues in -1 and -3 positions.

The triangulated solvent-accessible surface for each PDZ structure was built using MSMS software (Sanner et al., 1996) with a spherical probe radius of 1.4 Å and vertex density 10 Å ${}^{-1}$ . The largest connected set of surface vertices within 9 Å from the centre of the binding site was used to construct shape-based numerical descriptors. The descriptors are 508-dimensional vectors of non-negative integer numbers and were built using a shape distributions approach (Osada et al., 2002). In total 10 (Pawson, 2007) vertex triplets were selected randomly, each forming a triangle. Triangles which had a side longer than 16 Å were discarded. Triangle sides were distributed into 16 length bins, each 1 Å wide, covering lengths from 0 to 16 Å. A combination of three sorted side lengths, each belonging to 1 of 16 distance bins, defines 1 of 508 categories of the triangles. The number of triangles of each category was calculated, resulting in a 508-dimensional vector which is used as a numerical descriptor of the binding pocket shape. For further operations with descriptors, an Euclidian metric was introduced. Shape descriptors were distributed into 6 clusters using the k -means algorithm (Jain and Dubes, 1988). For each cluster, a centroid structure was defined as the one whose descriptor is the closest to mean descriptor for the cluster. The centroid structures 2O2T#B.pdb, 1VA8#3.pdb, 2DLU#01.pdb, 1UHP#8.pdb, 2OS6#8.pdb, and 3LNX#A.pdb were used for docking.

### 3.2 PDZ targeted library design

Screening collection by Enamine Ltd. (Chuprina et al., 2010), containing a total of 1 195 395 drug-like compounds, was used as the primary source of small molecules. The natural ligand of PDZ is the C-terminus of a peptide with carboxylic group making an extensive hydrogen-bond network with the “ Φ G Φ ” motif. Since the carboxyl group provides either a negative charge or a hydrogen-bond acceptor, we want our ligands to retain at least one of these features. Therefore, we pre-filtered the stock library to bear chemical groups which have negatively charged and/or hydrogen-bond acceptor functionality. In total 65 288 compounds were selected for the virtual screening study. The selected six centroids of PDZ domains and the prepared compound set were subjected to high-throughput docking using the QXP/Flo software (McMartin and Bohacek, 1997). Complexes were generated with 100 steps of sdock + routine, and 10 conformations per complex were saved.

Processing of docking poses started with filtering by contact term *Cntc* from the QXP/Flo scoring function. Entries with Cntc <45 were discarded, which removed complexes with weak geometries of bound ligands. The remained filtering was performed with the in-house MultiFilter program that allows for flexible geometry-based filtering. We applied two algorithms, *nearest-atom* filter and *hydrogen-bond* filter. The former filters complexes by distance from a given protein atom to the nearest heavy ligand atom, while in the latter filtering is based upon the number of hydrogen bonds calculated for a given complex geometry. With the *nearest-atom* routine we selected compounds that filled the P ${}_{0}$ pocket and sterically mimicked binding of a peptide carboxylic group. Peptide group hydrogens of the “ Φ G Φ ” motif and atoms forming the hydrophobic pocket were used for that. With the *hydrogen-bond* filter we selected compounds that formed extensive hydrogen bonding with the PDZ domain. Both these properties might have larger impact on binding rather than negative charge (Harris et al., 2003). Details on atoms used for filtering and thresholds for *hydrogen-bond* filtering, as well as the resulting number of compounds, are provided in Table S5. Compounds from complexes which passed through these filters were incorporated into a targeted library for the PDZ-domain family. The final library contained 1119 compounds in total.

### 3.3 Screening of compounds

Two-dimensional ${}^{1}H{-}^{15}N$ HSQC spectra were used to screen a library of 212 compounds designed by the company Enamine for PDZ domains. 50 µM of ${}^{15}N$ -labelled protein samples was prepared in a 20 mM sodium phosphate buffer, containing 50 mM sodium chloride, 0.02 % ( w/v ) ${\mathrm{NaN}}_{3}$ , at pH 7.4. Stock solutions of small molecules were prepared in DMSO-*d6* at a concentration of 160 mM. A ${}^{1}H{-}^{15}N$ HSQC spectrum of Dvl PDZ was acquired at 300 K with 5 % DMSO-*d6* in the absence of ligand as reference spectrum. Mixtures of 16 compounds were added to ${}^{15}N$ -labelled Dvl PDZ at eightfold molar excess each. The final concentration of DMSO-*d6* in the protein–ligand solutions was 5 %. Spectra were acquired with eight scans and 256 points in the indirect dimension. Compound binding was deduced if the resonance position of a cross peak was significantly shifted compared to the reference spectrum. The active compound was obtained through successive deconvolution. Experiments were recorded on a Bruker DRX600 spectrometer equipped with a triple-resonance cryoprobe. The preparation of samples was done automatically by a Tecan Genesis RSP 150 pipetting robot. Spectra were analysed using the programs TOPSPIN and SPARKY (Goddard and Kneller, 2003).

### 3.4 Synthesis of compounds

All reagents and starting materials were purchased from Sigma-Aldrich Chemie, ABCR, Alfa Aesar, or Acros Organics and used without further purification. All air or moisture-sensitive reactions were carried out under dry nitrogen using standard Schlenk techniques. Solvents were removed by evaporation on a Heidolph Laborota 4000 with vacuum provided by a PC 3001 Vaccubrand pump. Thin-layer chromatography (TLC) was performed on plastic-backed plates pre-coated with silica gel 60 F ${}_{254}$ (0.2 mm). Visualization was achieved under an ultraviolet (UV) lamp (254 and 366 nm). Flash chromatography was performed using J.T Baker silica gel 60 (30–63 µm ). Analytical high-performance liquid chromatography (HPLC) was performed on a Shimadzu LC-20 (degasser DGU-20A3, controller CBM-20A, autosampler SIL-20A) with a DAD-UV detector (SPD-M20A), using a reverse-phase C18 column (Nucleodur 100-5, 5 µM , 250 mm ×4 mm, Macherey-Nagel, Düren, Germany). Separation of compounds by preparative HPLC was performed on a Shimadzu LC-8A system equipped with a UV detector (SPD-M20A), using a semi-preparative C18 column (Nucleodur 100-5, 5 µM , 250 mm ×10 mm, Macherey-Nagel) or preparative C18 column (Nucleodur 100-5, 5 µM , 250 mm ×21 mm, Macherey-Nagel). The detection wavelength was 254 nm. Gradients of acetonitrile–water with 0.1 % TFA were used for elution at flow rates of 1, 8, and 14 mL min ${}^{-1}$ on the analytical, semi-preparative, and preparative columns, respectively. Melting point (mp) values were determined with a Stuart melting point apparatus, SMP3, and are not corrected. Mass spectra were recorded on a 4000Q TRAP LC–MS–MS system for AB Applied Biosystems MDS SCIEX. NMR spectra were recorded on a Bruker AV300 spectrometer instrument operating at 300 MHz for proton frequency using DMSO-*d6* solutions. Chemical shifts were quoted relative to the residual DMSO peak ( ${}^{1}H$ : δ=2.50 ppm, ${}^{13}C$ : δ=39.52 ppm). Coupling constants (J) are given in hertz (Hz). Splitting patterns are indicated as follows: singlet (s), doublet (d), triplet (t), quartet (q), multiple (m), and broad (b). Purity of each compound used for biological testing was ≥95 % unless otherwise noted. The purity checks of known inhibitors purchased for comparison with our compounds are found in Fig. S9.

#### 3.4.1 Synthesis of compounds **8**, **11**–**17**

To a solution of anthranilic acid substituted with the appropriate R ${}_{1}$ (1.32 mmol) and sodium carbonate (3.17 mmol) in water (2 mL) at 80 ${}^{\circ }$ C, the sulfonyl chloride (1.58 mmol) substituted with the appropriate R ${}_{2}$ was added over a period of 5 min. The stirring continued for 18 h at 80 ${}^{\circ }$ C. The reaction mixture was cooled to room temperature and acidified with 6 N HCl, and the resulting solid precipitate was filtered, washed with water, and dried to give the crude product. The final product was obtained by preparative HPLC (Puranik et al., 2008).

**Scheme 3:**
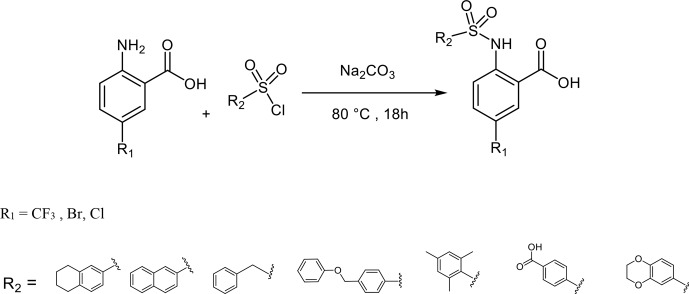
Synthesis of compounds **8**, **11**–**17**.

#### 3.4.2 2-(5,6,7,8-Tetrahydronaphthalene-2-sulfonamido)-5-(trifluoromethyl) benzoic acid (8)

(0.52 g, 74 % yield) ${}^{1}$ **H-NMR** (300 MHz, DMSO-d6): δ=11.77 [s, 1H, COOH], 8.13 [s, 1H, NH], 7.85 [d, ${}^{4}$ J ${}_{6,4}=2.1$ Hz, 1H, 6-H ${}_{\mathrm{Ar}}$ ] 7.62 [d, ${}^{4}$ J ${}_{1{}^{\prime },3{}^{\prime }}=2.1$ Hz, 1H, 1'-H ${}_{\mathrm{Ar}}$ ] 7.53 [dd, ${}^{3}$ J ${}_{4,3}=7.1$ Hz, ${}^{4}$ J ${}_{4,6}=2.1$ Hz, 4-H ${}_{\mathrm{Ar}}$ ] 7.36 [dd, ${}^{3}$ J ${}_{3{}^{\prime }4{}^{\prime }}=7.5$ Hz, ${}^{4}$ J ${}_{3{}^{\prime },1{}^{\prime }}=2.4$ Hz, 1H, 3'-H ${}_{\mathrm{Ar}}$ ] 7.15 [d, ${}^{3}$ J ${}_{4{}^{\prime },3{}^{\prime }}=7.5$ Hz, 1H, 4'-H ${}_{\mathrm{Ar}}$ ], 6.90 [d, ${}^{3}$ J ${}_{3,4}=7.1$ Hz, 1H, 3-H ${}_{\mathrm{Ar}}$ ] 2.73 (m, 4H, CH ${}_{2}$ ); 1.6 (m, 4H, CH ${}_{2}$ ). ${}^{13}$ **C-NMR** (75 MHz, DMSO-d6): δ=169.1 (C, C ${}_{\mathrm{Ar}}$ -8], 152.7(C, C ${}_{\mathrm{Ar}}$ -2), 143.8 (C, C ${}_{\mathrm{Ar}}$ -4a'), 138.7(C, C ${}_{\mathrm{Ar}}$ -2'), 135.9 (C, C ${}_{\mathrm{Ar}}$ -8a'), 130.4(CH, C ${}_{\mathrm{Ar}}$ -4), 128.7 (CH, C ${}_{\mathrm{Ar}}$ -6), 127.5 (CH, C ${}_{\mathrm{Ar}}$ -1'), 124.0 (CH, C ${}_{\mathrm{Ar}}$ -4'), 121.6 (C, C-6), 118.2 (C, C ${}_{\mathrm{Ar}}$ -5), 116.9 (C, C ${}_{\mathrm{Ar}}$ -3), 29.0 (CH ${}_{2}$ , C-8'), 28.8 (CH ${}_{2}$ , C-5'), 22.3 (CH ${}_{2}$ , C-6'), 22.2 (CH ${}_{2}$ , C-7'); mp: 177 ${}^{\circ }$ C; MS (ESI) m/z : calcd for C ${}_{18}$ H ${}_{16}$ F ${}_{3}$ NO ${}_{4}$ S, 399; found, 400 [M + H] ${}^{+}$ .

#### 3.4.3 5-Bromo-2-(naphthalene-2-sulfonamido) benzoic acid (11)

(0.13 g, 67 % yield) ${}^{1}$ **H-NMR** (300 MHz, DMSO-d6): δ=10.2 [s, 1H, COOH], 9.8 [s, 1H, NH] 8.59 [d, ${}^{4}$ J ${}_{1{}^{\prime },3{}^{\prime }}=1.4$ Hz, 1H, 1'-H ${}_{\mathrm{Ar}}$ ], 8.17 [d, ${}^{3}$ J ${}_{8{}^{\prime },7{}^{\prime }}=7.8$ Hz, 1H, 8'-H ${}_{\mathrm{Ar}}$ ], 8.10 [d, ${}^{3}$ J ${}_{4{}^{\prime }3{}^{\prime }}=8.8$ Hz, 1H, 4'-H ${}_{\mathrm{Ar}}$ ], 8.02 [d, ${}^{3}$ J ${}_{5{}^{\prime },6{}^{\prime }}=7.8$ Hz, 1H, 5'-H ${}_{\mathrm{Ar}}$ ], 7.93 [d, ${}^{4}$ J ${}_{6,4}=2.4$ Hz, 1H, 6-H ${}_{\mathrm{Ar}}$ ], 7.77 [dd, ${}^{3}$ J ${}_{3{}^{\prime },4{}^{\prime }}=8.8$ Hz, ${}^{4}$ J ${}_{3{}^{\prime },1{}^{\prime }}=1.4$ Hz, 1H, 3'-H ${}_{\mathrm{Ar}}$ ], 7.72–7.65 [m, 3H, 4-H ${}_{\mathrm{Ar}}$ , 6'-H ${}_{\mathrm{Ar}}$ , 7'-H ${}_{\mathrm{Ar}}$ ], 7.51 [d, ${}^{3}$ J ${}_{3,4}=8.9$ Hz, 1H, 3-H ${}_{\mathrm{Ar}}$ ]. ${}^{13}$ **C-NMR** (75 MHz, DMSO-d6): δ=168.2 (C, C-7), 138.8 (C, C ${}_{\mathrm{Ar}}$ -2), 136.8 (CH, C ${}_{\mathrm{Ar}}$ -4), 135.3 (C, C ${}_{\mathrm{Ar}}$ -4a'), 134.4 (C, C ${}_{\mathrm{Ar}}$ -8a'), 133.4 (CH, C ${}_{\mathrm{Ar}}$ -6), 131.4 (CH, C ${}_{\mathrm{Ar}}$ -6'), 129.3 (CH, C ${}_{\mathrm{Ar}}$ -4'), 128.5 (CH, C ${}_{\mathrm{Ar}}$ -8'), 127.8 ( 2×CH , C ${}_{\mathrm{Ar}}$ -5', C ${}_{\mathrm{Ar}}$ -7') 121.6 (CH, C ${}_{\mathrm{Ar}}$ -3'), 120.6 (CH, C ${}_{\mathrm{Ar}}$ -3), 119.0 (C, C ${}_{\mathrm{Ar}}$ -1), 114.9 (C, C ${}_{\mathrm{Ar}}$ -5). mp: 199 ${}^{\circ }$ C; (ESI) m/z : calcd for C ${}_{17}$ H ${}_{11}$ BrNO ${}_{4}$ S ${}^{-}$ , 403.9560; found, 403.9613 [M - H] ${}^{-}$ .

#### 3.4.4 5-Bromo-2-(phenylmethylsulfonamido)benzoic acid (12)

(0.07 g, 42 % yield) ${}^{1}$ **H-NMR** (300 MHz, DMSO-d6): δ=10.57 [s, 1H, COO H ], 8.05 [d, ${}^{4}$ J ${}_{6,4}=2.4$ Hz, 1H, 6-H ${}_{\mathrm{Ar}}$ ], 7.75 [dd, ${}^{3}$ J ${}_{4,3}=8.9$ Hz, ${}^{4}$ J ${}_{4,6}=2.4$ Hz, 1H, H-4 ${}_{\mathrm{Ar}}$ ], 7.49 [d, ${}^{3}$ J ${}_{3,4}=8.9$ Hz, 1H, 3-H ${}_{\mathrm{Ar}}$ ], 7.33–7.28 [m, 3H, 3'-H ${}_{\mathrm{Ar}}$ , 5'-H ${}_{\mathrm{Ar}}$ ], 7.23–7.20 [m, 2H, 4'-H ${}_{\mathrm{Ar}}$ ], 5.75 [s, 1H, N H ], 4.72 [s, 2H, 1'-H]. ${}^{13}$ **C-NMR** (75 MHz, DMSO-d6): δ=168.3 (C, C-7), 139.9 (C, C ${}_{\mathrm{Ar}}$ -2), 137(CH, C ${}_{\mathrm{Ar}}$ -4), 133.4 (CH, C ${}_{\mathrm{Ar}}$ -6), 130.7 (CH, C ${}_{\mathrm{Ar}}$ -3'), 128.6 (C, C ${}_{\mathrm{Ar}}$ -2'), 128.4 (CH, C ${}_{\mathrm{Ar}}$ -5'), 128.3 (CH, C ${}_{\mathrm{Ar}}$ -4'), 119.5 (CH, C ${}_{\mathrm{Ar}}$ -3), 117.5 (C, C ${}_{\mathrm{Ar}}$ -1), 113.9 (C, C ${}_{\mathrm{Ar}}$ -5), 57.4 (CH ${}_{2}$ , C-1'). mp: 216 ${}^{\circ }$ C; (ESI) m/z : calcd for C ${}_{14}$ H ${}_{11}$ BrNO ${}_{4}$ S ${}^{-}$ , 367.9860; found, 367.9878 [M - H] ${}^{-}$ .

#### 3.4.5 5-Bromo-2-(4-(phenoxymethyl)phenylsulfonamido)benzoic acid (13)

(0.6 g, 29 % yield) ${}^{1}$ **H-NMR** (300 MHz, DMSO-d6): δ=7.97 [d, ${}^{4}$ J ${}_{6,4}=2.4$ Hz, 1H, 6-H ${}_{\mathrm{Ar}}$ ), 7.85 (d, ${}^{3}$ J ${}_{2{}^{\prime },3{}^{\prime }}=8.3$ Hz, 2H, 3'-H ${}_{\mathrm{Ar}}$ ), 7.73 [dd, ${}^{3}$ J ${}_{4,3}=8.9$ Hz, ${}^{4}$ J ${}_{4,6}=2.4$ Hz, 1H, 4-H ${}_{\mathrm{Ar}}$ ], 7.63 [d, ${}^{3}$ J ${}_{2{}^{\prime },3{}^{\prime }}=8.3$ Hz, 2H, 2'-H ${}_{\mathrm{Ar}}$ ], 7.47 [d, ${}^{3}$ J ${}_{3,4}=8.9$ Hz, 1H, 3-H ${}_{\mathrm{Ar}}$ ], 7.29 [dd, ${}^{3}$ J ${}_{3{}^{\prime \prime },2{}^{\prime \prime }}{=}^{3}$ J ${}_{3{}^{\prime \prime },4{}^{\prime \prime }}=7.3$ Hz, 2H, 3”-H ${}_{\mathrm{Ar}}$ ], 7.00–6.92 [m, 3H, 4”-H ${}_{\mathrm{Ar}}$ , 2”-H ${}_{\mathrm{Ar}}$ ], 5.17 [s, 2H, 5'-H]. ${}^{13}$ **C-NMR** (75 MHz, DMSO-d6): δ=168.2 (C, C-7), 157.9 (C, C ${}_{\mathrm{Ar}}$ -1”), 143.2 (C, C ${}_{\mathrm{Ar}}$ -4'), 138.8 (C, C ${}_{\mathrm{Ar}}$ -2), 137.5 (C, C ${}_{\mathrm{Ar}}$ -1'), 136.9 (CH, C ${}_{\mathrm{Ar}}$ -4) 133.5 (CH, C ${}_{\mathrm{Ar}}$ -6), 129.4(CH, C ${}_{\mathrm{Ar}}$ -3”), 128.1(CH, C ${}_{\mathrm{Ar}}$ -2'), 127.0 (CH, C ${}_{\mathrm{Ar}}$ -3'), 120.9 (CH, C ${}_{\mathrm{Ar}}$ -4”), 120.5 (CH, C ${}_{\mathrm{Ar}}$ -3), 119.0 (C, C ${}_{\mathrm{Ar}}$ -1), 114.9(CH, C ${}_{\mathrm{Ar}}$ -5), 114.7 (CH, C ${}_{\mathrm{Ar}}$ -2”), 68.0 (CH ${}_{2}$ , C-5') mp: 175 ${}^{\circ }$ C; (ESI) m/z : calcd for C ${}_{20}$ H ${}_{15}$ BrNO ${}_{5}$ S ${}^{-}$ , 459.9860; found, 459.9878 [M - H] ${}^{-}$ .

#### 3.4.6 5-Bromo-2-(2,4,6-trimethylphenylsulfoamido)benzoic acid (14)

(0.6 g, 78 % yield) ${}^{1}$ **H-NMR** (300 MHz, DMSO-d ${}_{6})$ : δ=11.77 [s, 1H, COOH], 9.98 [s, 1H, NH], 7.68 [d, ${}^{4}$ J ${}_{6,4}=2.4$ Hz, 1H, 6-H ${}_{\mathrm{Ar}}$ ], 7.51 [dd, ${}^{3}$ J ${}_{4,3}=7.1$ Hz, ${}^{4}$ J ${}_{4,6}=2.4$ Hz, 1H 4-H ${}_{\mathrm{Ar}}$ ], 7.17 [d, 2H, 4'-H ${}_{\mathrm{Ar}}$ , 6'-H ${}_{\mathrm{Ar}}$ ], 7.14 [d, ${}^{3}$ J ${}_{3,4}=$ 1H, 3-H ${}_{\mathrm{Ar}}$ ], 2.56 [s, 6H, CH ${}_{3}$ , 9'-H, 7'-H], 2.21 [s, 3H, CH ${}_{3}$ , 8'-H]. ${}^{13}$ **C-NMR** (300 MHz, DMSO-d ${}_{6})$ : δ=168.8 (C, C-7), 143.3 (C, C ${}_{\mathrm{Ar}}$ -2), 139.5 (C, C ${}_{\mathrm{Ar}}$ -2'), 139.0 and 139.0 ( 2× C, C ${}_{\mathrm{Ar}}$ -3', C ${}_{\mathrm{Ar}}$ -1') 137.3 (CH, C ${}_{\mathrm{Ar}}$ -4), 134.0 (CH, C ${}_{\mathrm{Ar}}$ -6'), 133.0 (CH, C ${}_{\mathrm{Ar}}$ -6), 132.5 and 132.5 ( 2× CH, C ${}_{\mathrm{Ar}}$ -4', C ${}_{\mathrm{Ar}}$ -6') 119.1 (CH, C ${}_{\mathrm{Ar}}$ -3), 117.9 (C, C ${}_{\mathrm{Ar}}$ -5), 114.3 (C, C ${}_{\mathrm{Ar}}$ -1), 22.5 and 22.5 ( 2× CH ${}_{3}$ , C-7', C-9') 20.7 (CH ${}_{3}$ , C-8'); mp: 185; MS (ESI): m/z : calcd for C ${}_{16}$ H ${}_{16}$ BrNO ${}_{4}$ S, 397; found, 398 [M + H] + .

#### 3.4.7 2-(4-Acetylphenylsulfoamido)-5-(trifluoromethyl)benzoic acid (15)

(0.4 g, 63 % yield) ${}^{1}$ **H-NMR** (300 MHz, DMSO-d6): δ=12.28 [s, 1H, COOH]; 12.10 [s, 1H, NH], 8.11 [d, ${}^{4}$ J ${}_{6,4}=2.5$ Hz, 1H, 6-H ${}_{\mathrm{Ar}}$ ], 8.08 [d, ${}^{3}$ J ${}_{3{}^{\prime }2{}^{\prime }}=7.5$ Hz, 2H, 3'-H ${}_{\mathrm{Ar}}$ ], 7.86 [dd, ${}^{4}$ J ${}_{4,6}=2.5$ Hz, ${}^{3}$ J ${}_{4,3}=7.3$ Hz, 1H, 4-H ${}_{\mathrm{Ar}}$ ], 7.64 [d, ${}^{3}$ J ${}_{4,3}=7.3$ Hz, 1H, 3-H ${}_{\mathrm{Ar}}$ ], 7.56 [dd ${}^{3}$ J ${}_{2{}^{\prime },3{}^{\prime }}=7.5$ Hz, ${}^{4}$ J ${}_{2{}^{\prime },6{}^{\prime }}=2.3$ Hz, 2H, 2'-H ${}_{\mathrm{Ar}}$ , 6'-H ${}_{\mathrm{Ar}}$ ] 7.22 [dd, ${}^{3}$ J ${}_{3{}^{\prime },2{}^{\prime }}=7.5$ Hz, ${}^{4}$ J ${}_{3{}^{\prime },5{}^{\prime }}=2.1$ Hz, 2H, 3'-H ${}_{\mathrm{Ar}}$ , 5'-H ${}_{\mathrm{Ar}}$ ] 2.50 [s, 3H, CH ${}_{3}$ , 8'-H]. ${}^{13}$ **C-NMR** (75 MHz, DMSO-d6): δ=197.9 (C, C-7'), 169.1 (C, C-8), 151.8 (C, C ${}_{\mathrm{Ar}}$ -2) 143.5 (C, C ${}_{\mathrm{Ar}}$ -1'), 142.5 (C, C ${}_{\mathrm{Ar}}$ -4'), 140.6 (CH, C ${}_{\mathrm{Ar}}$ -4), 131.4 (CH, C ${}_{\mathrm{Ar}}$ -7), 129.6 ( 2× CH, C ${}_{\mathrm{Ar}}$ -3', C ${}_{\mathrm{Ar}}$ -5'), 128.6 ( 2× CH, C ${}_{\mathrm{Ar}}$ -2', C ${}_{\mathrm{Ar}}$ -6'), 127.6 (C, C ${}_{\mathrm{Ar}}$ -6), 123.0 (C, C- ${}_{\mathrm{Ar}}$ -5), 118.7 (CH, C ${}_{\mathrm{Ar}}$ -3), 27.3 (CH ${}_{3}$ , C-8'); mp: 170 ${}^{\circ }$ C; MS (ESI) m/z : calcd for C ${}_{16}$ H ${}_{12}$ F ${}_{3}$ NO ${}_{5}$ S, 387; found, 388 [M + H] ${}^{+}$ .

#### 3.4.8 2-(2,3-Dihydrobenzo[ b ][1,4]dioxine-6-sulfonamido)-5-(trifluoromethyl)benzoic acid (16)

(0.4 g, 65 % yield) ${}^{1}$ **H-NMR** (300 MHz, DMSO-d6): δ=11.48 [s, 1H, COOH], 8.13 [s, 1H, NH], 7.89 [d, ${}^{4}$ J ${}_{6,4}=2.5$ Hz, 1H, 6-H ${}_{\mathrm{Ar}}$ ] 7.66 [dd, ${}^{3}$ J ${}_{4,3}=8.1$ Hz, ${}^{4}$ J ${}_{4,6}=2.5$ Hz, 1H, 4-H ${}_{\mathrm{Ar}}$ ], 7.23 [d, ${}^{3}$ J ${}_{4,3}=8.1$ Hz 1H, 3-H ${}_{\mathrm{Ar}}$ ], 7.11 [dd, ${}^{3}$ J ${}_{2{}^{\prime },3{}^{\prime }}=7.3$ Hz, ${}^{4}$ J ${}_{2{}^{\prime },8{}^{\prime }}=3.2$ Hz, 1H, 2'-H ${}_{\mathrm{Ar}}$ ] 6.95 [ d, ${}^{4}$ J ${}_{2{}^{\prime },8{}^{\prime }}=3.2$ Hz, 1H, 8'-H ${}_{\mathrm{Ar}}$ ] 4.23–4.31 [m, 4H, 5'-H, 6'-H]. ${}^{13}$ **C-NMR** (75-MHz, DMSO-d6): δ=168.9 (C, C-8), 148.3 (C, C-4'), 143.8 (C, C-2), 143.5 (C, C-7'), 131.3 (C, C-1'), 130.8 (CH, C-4), 128.6 (CH, C-6), 125.7 (C, C-7), 122.1 (C, C-5), 120.9 (CH, C-2'), 118.3 (CH, C-3), 118.1 (CH, C-3'), 116.8 (CH, C-8'), 64.7 (CH ${}_{2}$ , C-5') 64.3 (CH ${}_{2}$ , C-6'); mp: 178 ${}^{\circ }$ C; MS (ESI) m/z : calcd for C ${}_{16}$ H ${}_{12}$ F ${}_{3}$ NO ${}_{6}$ S, 403; found, 404 [M + H] ${}^{+}$ .

#### 3.4.9 5-(Trifluoromethyl)-2-(2,4,6-trimethylphenylsulfoamido)benzoic acid (17)

(0.38 g, 62 % yield) ${}^{1}$ **H-NMR** (300 MHz, DMSO-d6): δ=12.28 [s, 1H, COOH], 11.60 [s, 1H, NH], 8.15 [d, ${}^{4}$ J ${}_{6,4}=2.1$ Hz, 1H, 6-H ${}_{\mathrm{Ar}}$ ] 7.92 [dd, ${}^{3}$ J ${}_{4,3}=7.9$ Hz, ${}^{4}$ J ${}_{4,6}=2.1$ Hz, 1H, 4-H ${}_{\mathrm{Ar}}$ ] 7.87 [d, ${}^{4}$ J ${}_{6{}^{\prime },4{}^{\prime }}=1.9$ Hz, 2H, 4'-H ${}_{\mathrm{Ar}}$ , 6'-H ${}_{\mathrm{Ar}}$ ], 7.48 [d, ${}^{3}$ J ${}_{3,4}=7.9$ Hz, 1H, 3-H ${}_{\mathrm{Ar}}$ ], 2.60 [s, 6H, CH ${}_{3}$ , 9'-H, 7'-H], 2.23 [s, 3H, CH ${}_{3}$ , 8'-H]. ${}^{13}$ **C-NMR** (75 MHz, DMSO-d6): δ=169.3 (C, C-7), 154.2 (C, C-2), 143.6 (C, C-2') , 139.1 and 139.1 ( 2× C, C-1', C-3') 132.9 (C, C-5'), 132.5 (CH, C-4), 131.5 and 131.5 ( 2× CH, C-4', C-6'), 130.1(CH, C-6), 128.7 (C, C-8), 122.5 (C, C-5), 117.0 (CH, C-3), 109.0 (C, C-1), 22.4 and 22.4 ( 2× CH ${}_{3}$ , C-7', C-9'), 20.8 (CH ${}_{3}$ , C-8'); mp: 184 ${}^{\circ }$ C; MS (ESI) m/z : calcd for C ${}_{17}$ H ${}_{16}$ F ${}_{3}$ NO ${}_{4}$ S, 387; found, 388 [M + H] ${}^{+}$ .

**18**, **19**, **20**, and **21** were purchased from Enamine, Kiev, Ukraine, as pure compounds (see also Table S6).

### 3.5 Determination of ligand binding and binding constant by NMR

50 µM of ${}^{15}N$ -labelled protein samples was prepared in a 20 mM sodium phosphate buffer containing 50 mM sodium chloride, 0.02 % ( w/v ) ${\mathrm{NaN}}_{3}$ , at pH 7.4. Stock solutions of small molecules were prepared in DMSO-*d6* at a concentration of 160 mM. A ${}^{1}H{-}^{15}N$ HSQC spectrum of Dvl PDZ was acquired at 300 K with 5 % DMSO-*d6* in the absence of ligand as reference spectrum. Mixtures of 16 compounds were added to ${}^{15}N$ -labelled Dvl PDZ at eightfold molar excess each. The final concentration of DMSO-*d6* in the protein–ligand solutions was 5 %. Spectra were acquired with eight scans and 256 points in the indirect dimension.

Binding was deduced if the resonance position of a cross peak was significantly shifted compared to the reference spectrum. The active compound was obtained through successive deconvolution. Experiments were recorded on a Bruker DRX600 spectrometer equipped with a triple-resonance cryoprobe. The preparation of samples was done automatically by a Tecan Genesis RSP 150 pipetting robot. Spectra were analysed using the programs TOPSPIN and SPARKY.

Chemical shift perturbations were obtained by comparing the ${}^{1}H{-}^{15}N$ backbone resonances of protein alone to those of protein–ligand complex. The mean shift difference ( Δδ in ppm) was calculated using Eq. (1) (Garrett et al., 1997; Bertini et al., 2011).

1 $$\Delta \delta =\sqrt{\frac{[\Delta \delta_{H}{]}^{2}+[\Delta \delta_{N}/5{]}^{2}}{2}}$$

Here $\Delta \delta_{N}$ and $\Delta \delta_{H}$ are the amide nitrogen and amide proton chemical shift differences between the free and the bound states of the protein. In order to estimate binding constants, titration experiments monitored by NMR were done. A series of ${}^{1}H{-}^{15}N$ HSQC were recorded as a function of ligand concentration. Residues showing a continuous chemical shift change and for which the intensity remained strong were classified as being in fast exchange. The dissociation binding constant was estimated by fitting the observed chemical shift change to Eq. (2) (Shuker et al., 1996; Hajduk et al., 1997).

2 $$\begin{array}{rl} & \frac{\Delta \delta }{\Delta \delta_{max}}\\ & =\frac{\left(\left[L_{T}\right]+\left[P_{T}\right]+K_{D}\right)-\sqrt{{\left([L_{T}]+[P_{T}]+K_{D}\right)}^{2}-4[L_{T}]\cdot [P_{T}]}}{2[P_{T}]} \end{array}$$

Δδ is the observed protein amide chemical shift change at a given compound concentration, and $\Delta \delta_{max}$ is the maximum chemical shift change at saturation. $\left[L_{T}\right]$ is the total concentration of the compound, and $\left[P_{T}\right]$ is the total concentration of the protein. K ${}_{D}$ is the equilibrium dissociation constant. The K ${}_{D}$ values are reported as means ± standard deviations of at least six residues influenced upon binding of the ligand.

### 3.6 Determination of binding constant by isothermal titration calorimetry (ITC)

Isothermal titration calorimetry (ITC) experiments were performed using a VP-ITC system (MicroCal). Protein in 20 mM HEPES buffer and 50 mM NaCl (pH 7.4) was centrifuged and degassed before the experiment. A 200 µM ligand solution containing 2 % DMSO was injected 30 times in 10 µL aliquots at 120 s intervals with a stirring speed of 1000 rpm into a 1.4 mL sample cell containing the Dvl PDZ domain at a concentration of 20 µM at 25 ${}^{\circ }$ C. Control experiment was initially determined by titrating ligand into buffer at the same conditions. Titration of ligand into buffer yielded negligible heat. Thermodynamic properties and binding constants were determined by fitting the data with a non-linear least-squares routine using a single-site binding model with Origin (OriginLab) for ITC v.7.2 (MicroCal).

### 3.7 Protein expression

PDZ domains of human AF6 (P55196-2, residues 985–1086) and murine α 1-syntrophin (Q61234, residues 81–164) were cloned into pGEX-6P-2 (Amersham Biosciences, Freiburg, Germany) and pGAT2 (European Molecular Biology Laboratory, Heidelberg, Germany), respectively. Proteins were expressed in *E. coli* BL21 (DE3) cells and purified as previously described (Boisguerin et al., 2004). For the cloning of the Dvl-1 PDZ domain (O14640, residues 245–338), IMAGp958J151157Q (ImaGenes) was used as template. V250 is exchanged to isoleucine as in human Dvl-3 or murine Dvl-1. The C-terminal C338 of the domain was exchanged by serine. Via cloning in pET46EK/LIC, a coding sequence for a TEV (tobacco etch virus) protease cleavage site was introduced. The resulting plasmid, pDVL1, was transformed in *E. coli* BL21 (DE3). Expression on twofold M9 minimal medium with 0.5 g L ${}^{-1}$ ${}^{15}N$ ${\mathrm{NH}}_{4}\mathrm{Cl}$ as sole nitrogen source in shaking culture was done at 25 ${}^{\circ }$ C overnight with 1 mM IPTG. A yield of 25 mg of pure Dvl-1 was obtained from 1 L culture after IMAC, TEV protease cleavage, a second IMAC, and gel filtration (Superdex 75). The protein domain Dvl-1_245–338 was supplied for NMR in 20 mM phosphate buffer, pH 7.4, and 50 mM NaCl.

The production of Dvl-3 (Q92997 residues 243–336), mShank3 (Q4ACU6, residues 637–744) PDZ domains and the three PDZ domains of PSD95 was described by Saupe et al. (2011).

### 3.8 Crystallization and X-ray diffraction

The His-tagged cleaved human Dvl-3 PDZ domain was concentrated to 12–20 mg mL ${}^{-1}$ in the presence of a fivefold molar excess of compounds **3**, **5**, **6**, **7**, **11**, and **12**. Crystals of all complexes were grown at room temperature by the sitting drop vapour-diffusion method. 200 nL Dvl-3/compound solution was mixed with an equal volume of reservoir solution using the Gryphon (Formulatrix) pipetting robot. Crystals of all complexes were grown to their final size within 4 to 14 d. The Dvl-3 PDZ domain crystallized in complex with compounds **3** and **7** in crystallization condition 30 % PEG 8000, 0.2 M ammonium sulfate, 0.1 M MES pH 6.5; with compound **5** in 30 % PEG 8000, 0.1 M MES pH 5.5; with compound **6** in 1.2 M ammonium sulfate, 0.1 M citric acid pH 5.0; with compound **12** in 32 % PEG 8000, 0.2 M ammonium sulfate, 0.1 M Na-cacodylate pH 6.0; with compound **11** in 1 M ammonium sulfate, 1 % PEG 3350, 0.1 M Bis-Tris pH 5.5; with compound **12** in 1.26 M sodium phosphate, 0.14 M potassium phosphate; and with compound **18** in 1.5 M ammonium sulfate, 12 % glycerol, 0.1 M Tris-HCl pH 8.5. The crystals were cryoprotected if necessary, for data collection by soaking for a few seconds in precipitant solution containing 20 % ( v/v ) glycerol and subsequently frozen in liquid nitrogen. Diffraction data were collected at 100 K at beamline BL14.1 at the synchrotron radiation source BESSY, Helmholtz-Zentrum Berlin, and processed with XDS.

### 3.9 Structure determination and refinement

Phases for the Dvl-3 PDZ domain in complex with compound **3** were obtained by molecular replacement with PHASER (McCoy et al., 2007) using the *Xenopus laevis* Dishevelled PDZ-domain structure (PDB code 2F0A) as a starting model. The reasonable crystal packing and electron density allowed for further model and compound building using the program COOT (Emsley and Cowtan, 2004) with iterative refinement with REFMAC (Murshudov et al., 1997). All further complex structures were obtained in the same way but using the final refined compound-free Dvl-3-PDZ structure as model for molecular replacement. The Ramachandran statistics were analysed by MolProbity (Chen et al., 2010) for all complexes, and all crystallographic statistics are given in Tables S2 and S3. Figures were prepared with PyMOL. Atomic coordinates and structure factor amplitudes for DVL-3 PDZ domain in complex with compounds **3**, **5**, **6**, **7**, **11**, **12**, **18** were deposited in the Protein Data Bank with accession codes 6ZBQ, 6ZBZ, 6ZC3, 6ZC4, 6ZC6, 6ZC7, and 6ZC8, respectively.

### 3.10 MTT assay

HEK293 cells were plated on a 96-well plate and treated with different concentrations of Dvl inhibitors. After 24 h treatment, 20 µL of MTT solution (5 mg mL ${}^{-1}$ ) was added into each well. After 2 h incubation, cell culture medium was replaced with 50 µL DMSO, and the signal of the purple formazan, produced by living cells, was measured by a plate reader.

### 3.11 TOP-GFP reporter assay

The lentivirus particle (CCS-018L, SABiosciences) encoding GFP under the control of a basal promoter element (TATA box) joined to tandem repeats of a consensus TCF/LEF binding site was transfected into HEK293 cells. Stable cells were selected by puromycin (2 $µg\;{\mathrm{mL}}^{-1}$ ) treatment. Wnt signalling activity indicated by GFP intensity was measured by flow cytometry after 24 h incubation with recombinant mouse Wnt3a (100 ng mL ${}^{-1}$ ) or GSK3 inhibitor CHIR99021 (3 µM ) in the presence of Dvl inhibitors.

### 3.12 Luciferase reporter assays

Plasmids encoding a firefly luciferase reporter gene under the control of different responsive elements were transfected into HeLa cells with a pRL-SV40 normalization reporter plasmid using the Lipofectamine 2000 (Invitrogen). After desired treatment, cells were harvested in the passive lysis buffer (Promega), and 15 µL cell lysate was transferred to 96-well LumiNunc plates (Thermo Scientific). Firefly luciferase and Renilla luciferase were detected with the D-luciferin buffer (75 mM HEPES, 4 mM MgSO ${}_{4}$ , 20 mM DTT, 100 µM EDTA, 0.5 mM ATP, 135 µM Coenzyme A, and 100 µM D-Luciferin sodium salt; pH 8.0) and the coelenterazine buffer (15 mM Na4PPi, 7.5 mM NaAc, 10 mM CDTA, 400 mM Na ${}_{2}$ SO ${}_{4}$ , 25 µM APMBT, and 1.1 µM coelenterazine; pH 5.0), respectively, using the CentroXS LB960 lumiometer (Berthold Technologies).

### 3.13 Immunoblotting

To assess the β -catenin accumulation in HeLa cells, cells were treated with Wnt3a in the presence of Dvl inhibitors for 24 h and lysed in RIPA buffer (50 mM Tris, pH 8.0, 1 % NP-40, 0.5 % deoxycholate, 0.1 % SDS, 150 mM NaCl). Equal amounts of protein were loaded on a SDS-PAGE. Separated proteins were blotted onto PVDF membranes (polyvinylidene fluoride) for immunoblot analysis using anti- β -catenin antibody (610154, BD). HRP-conjugated anti-mouse antibody (715-035-150, Jackson ImmunoResearch Laboratories) was used for secondary detection with Western lightning chemiluminescence reagent plus (PerkinElmer) and Vilber Lourmat imaging system SL-3.

### 3.14 qRT-PCR analysis

To measure the Wnt target accumulation at mRNA level, HeLa cells were treated with Wnt3a in the presence of Dvl inhibitors for 24 h. mRNA was extracted according to the standard TRIzol^®^ protocol (Invitrogen) and reverse-transcribed using random primers (Invitrogen) and M-MLV reverse transcriptase (Promega). The qRT-PCR was performed in a iQ5 multi-colour real-time PCR detection system (Bio-Rad) using SYBR^®^ Green (Thermo Scientific) and gene-specific primer pairs of Bmp2, Axin2, LEF1, and β -actin (endogenous control).

### 3.15 Migration assay

Cell motility was assessed using 24-well trans-well assay (pore diameter: 8 µm , Corning). SW480WL cells were seeded in the upper chamber in serum-free DMEM with 0.1 % BSA; 20 % serum was supplemented to medium in the lower chamber. After incubation with Wnt3a in the presence of Dvl inhibitors for 24 h, non-migrant cells were scraped off using a cotton swab; the migrated cells on the filters were stained with DAPI, photographed, and counted.

### 3.16 Colon sphere culture

SW480WL cells were trypsinized into single cells, seeded on 24-well cell culture plates, pre-coated with 250 µL poly-HEMA (12 mg mL ${}^{-1}$ in 95 % ethanol, Sigma) per well, and incubated with Wnt3a in the presence of Dvl inhibitors in the sphere culture medium (F12 : DMEM 1:1 , 1 × B-27 supplement, 20 ng mL ${}^{-1}$ EGF, 20 ng mL ${}^{-1}$ FGF, 0.5 % methylcellulose) for 10 d. Numbers of spheres were then counted under the microscope.

## 4 Conclusions

In the present work, small molecules that bind to Dvl PDZ in the one-digit micromolar range with considerable selectivity have been developed by an extensive structure-based design approach. With regards to the affinity determined by ITC, compound **18** binds to Dvl-1 and Dvl-3 in vitro with K ${}_{D}$ values of 2.4 and 9.4, respectively, comparing very well with known ligands. X-ray structures of Dvl-3 PDZ complexes with selected compounds provided insight into crucial interactions and served as the basis for the design of tight binding compounds with reduced toxicity. The structural investigations revealed that these compounds form hydrogen bonds with the amide groups of residues L260, G261, and I262 in the PDZ-domain loop and the side chains of residues H324 and R320. Finally, the chosen methodology, i.e. virtual screening followed by a two-stage NMR-based screening, X-ray crystallography, and chemical synthesis, is an excellent path towards bioactive interaction partners. Our best compounds effectively inhibited the canonical Wnt signalling pathway in a selective manner and could be developed for further studies.

## Supplement

10.5194/mr-2-355-2021-supplement The supplement related to this article is available online at: https://doi.org/10.5194/mr-2-355-2021-supplement.

## Data Availability

The underlying research data are available from the authors upon request by email.

## Code availability

Accession codes, atomic coordinates, and structure factor amplitudes for Dvl-3 PDZ domain in complex with compounds 3, 5, 6, 7, 11, 12, and 18 were deposited in the Protein Data Bank with accession codes 6ZBQ, 6ZBZ, 6ZC3, 6ZC4, 6ZC6, 6ZC7, and 6ZC8, respectively. Authors will release the atomic coordinates and experimental data upon article publication.

## Appendix

The supplement related to this article is available online at: https://doi.org/10.5194/mr-2-355-2021-supplement.
