# Supplementary material for: Small-molecule inhibitors of the PDZ domain of Dishevelled proteins interrupt Wnt signalling
Source: Magn Reson (Gott). 2021 Jun 2;2(1):355–74. doi: 10.5194/mr-2-355-2021 (PMC10539800; doi:10.5194/mr-2-355-2021)
Supplement: The supplement related to this article is available online at: https://doi.org/10.5194/mr-2-355-2021-supplement. [file mr-2-355-supplement.pdf]

Supplement of Magn. Reson., 2, 355–374, 2021
https://doi.org/10.5194/mr-2-355-2021-supplement
© Author(s) 2021. CC BY 4.0 License.

Supplement of

Small-molecule inhibitors of the PDZ domain of Dishevelled proteins interrupt Wnt signalling

Nestor Kamdem et al.

Correspondence to: Hartmut Oschkinat (oschkinat@fmp-berlin.de)

The copyright of individual parts of the supplement might differ from the article licence.

Tables of contents

1. Structure-based alignment of the amino acid sequences of Dvl-1,2,3 PDZ ; PSD95-PDZ-1,2,3 ; Af-6 and Syn PDZ domains. (S.2)

2. 1H-15N HSQC spectra of Dvl-3 PDZ domain alone and in the presence of varying concentrations of compound 3. (S.3)

3. Detailed views of diverse compounds bound to the Dvl-3 PDZ domain. (S.4)

4. Cell viability assays of compounds 3, 7,8, 9, 10, (A) and 18, 20, 21 (B). (S.5)

5. ITC binding assays of compound 18 with Dvl-3 PDZ (A) and with Dvl-1 PDZ (B). (S.5)

6. Structures of selected compounds used for comparison to our compounds. (S.6)

7. ITC data of selected compounds used for comparison to our compounds. (S.7)

8. NMR binding assay with compound 322338/3289-8625. (S.8)

9. Purity check of compounds. (S.9)

- Purity check of NPL-1011 compound. (S.9)
- Purity check of Sulindac compound. (S.10)
- Purity check of CalBioChem-322338 compound. (S.11)
- Purity check of NSC668036 compound. (S.12)
- LCMS of intermediate compound 8. (S.13)
- LCMS of intermediate compound 14. (S.13)

10. Chemical shift perturbation values of Dvl-3 PDZ and Dvl-1 PDZ for compounds (2-21). (S.14)

11. Data collection and refinement statistics of compounds 3, 5, 6, 7. (S.15)

12. Data collection and refinement statistics of compounds 11, 12, 18. (S.16)

13. Selectivity of ligands derived from chemical shift perturbation of compounds tested at other PDZ domains. (S.17)

14. Details of Multifilter routines. (S.17)

15. Smiles codes and Compounds ID. (S.18)

16. NMR characterization of synthesized compounds (8 , 11 , 13 , 14 , 15 , 16 , 17). (S.21)

1. Structure-based alignment of the amino acid sequences of Dvl-1,2,3 PDZ ; PSD95-PDZ-1,2,3 ; Af-6 and Syn PDZ domains.

```
hDVL1            TVTLNMERHH FLGISIVGQS N--DRGDG-- ------GIYI GSIMKGGAVA ADGRIEPGDM
hDVL2            TVTLNMEKYN FLGISIVGQS N--ERGDG-- ------GIYI GSIMKGGAVA ADGRIEPGDM
hDVL3            TVTLNMEKYN FLGISIVGQS N--ERGDG-- ------GIYI GSIMKGGAVA ADGRIEPGDM
PSD-95 PDZ1      EITLERGN-S GLGFSIAGGT DNPHIGDDP- ------SIFI TKIIPGGAAA QDGRLRVNDS
PSD-95 PDZ2      EIKLIKGP-K GLGFSIAGGV GNQHIPGDN- ------SIYV TKIIEGGAAH KDGRLQIGDK
PSD-95 PDZ3      RIVIHRGS-T GLGFNIVGG- -----EDGE- ------GIFI SFILAGGPAD LSGELRKGDQ
hAF6             -ITVTLKKQN GMGLSIVAAK G--AGQDKL- ------GIYV KSVVKGGAAD VDGRLAAGDQ
h_alpha_Syn PDZ  RVTVRKADAG GLGISIKG-- ---GRENKM- ------PILI SKIFKGLAAD QTEALFVGDA
mShank3 PDZ      VAILQKRDHE GFGFVLRGAK AETPIEEFTP TPAFPALQYL ESVDVEGVAW RAG-LRTGDF

hDVL1            LLQVNDVNFE NMSNDDAVRV LREIVSQTGP ISLTVAKCWD PT
hDVL2            LLQVNDMNFE NMSNDDAVRV LRDIVHKPGP IVLTVAKCWD PS
hDVL3            LLQVNEINFE NMSNDDAVRV LREIVHKPGP ITLTVAKCWD PS
PSD-95 PDZ1      ILFVNEVDVR EVTHSAAVEA LKEAGS---I VRLYVMRR-- --
PSD-95 PDZ2      ILAVNSVGLE DVMHEDAVAA LKNTYD---V VYLKVAKP-- --
PSD-95 PDZ3      ILSVNGVDLR NASHEQAAIA LKNAGQ---T VTIIAQYK-- --
hAF6             LLSVDGRSLV GLSQERAAEL MTRTSS---V VTLEVAKQG- --
h_alpha_Syn PDZ  ILSVNGEDLS SATHDEAVQV LKKTGK---E VVLEVKYMK- --
mShank3 PDZ      LIEVNGVNVV KVGHKQVVGL IRQGGN---R LVMKVVSVT- --
```

Figure S1: Structure-based alignment of the amino acid sequences of Dvl1,2 and 3 PDZ, Psd-1,2,3 PDZ, Af-6 and Syn PDZ domains. For Dvl PDZ, differences are highlighted in blue and similarities are highlighted in purple. UNIPROT codes: O14640 (Dvl-1 PDZ); O14641 (Dvl-2 PDZ); Q92997 (Dvl-3 PDZ), P78352 (Psd-1, Psd-2, Psd-3 PDZ); Q13424 (Alpha-1 Sytr PDZ); P55196 (Af6 PDZ); Q4ACU6 (mShank-3 PDZ)

2. 1H-15N HSQC spectra of Dvl-3 PDZ domain alone and in the presence of varying concentrations of compound 3

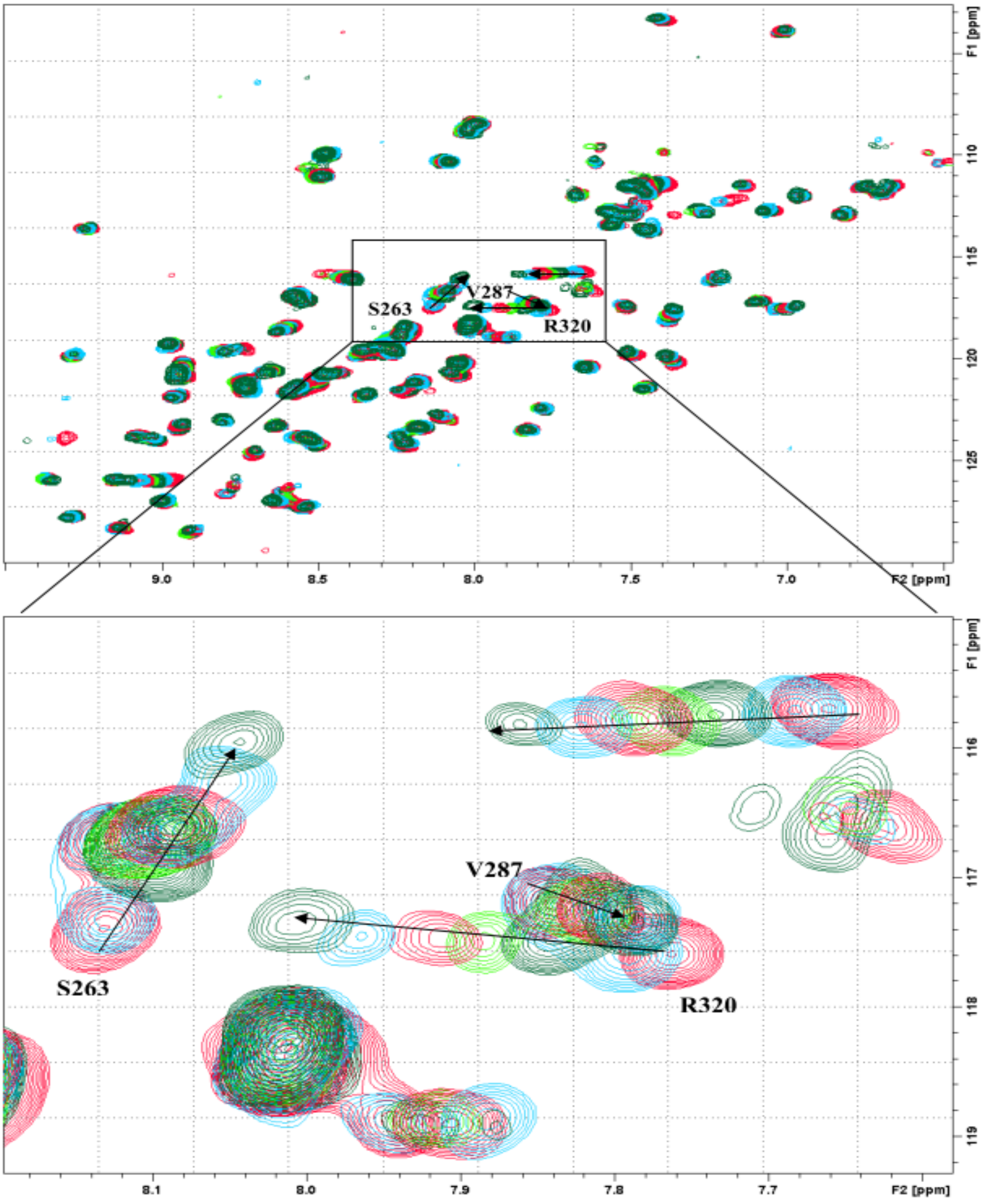

Figure S2: ^{1}H-^{15}N HSQC spectra of Dvl-3 PDZ domain alone (concentration of 50 μM) and in the presence of varying concentrations of compound **1** (25, 75, 100, 150, 200, 300, 400 μM) . The arrows indicate the gradual change of chemical shifts with increasing ligand concentration for residues surrounding the binding pocket of Dvl-3 PDZ.

3. Detailed views of diverse compounds bound to the Dvl-3 PDZ domain

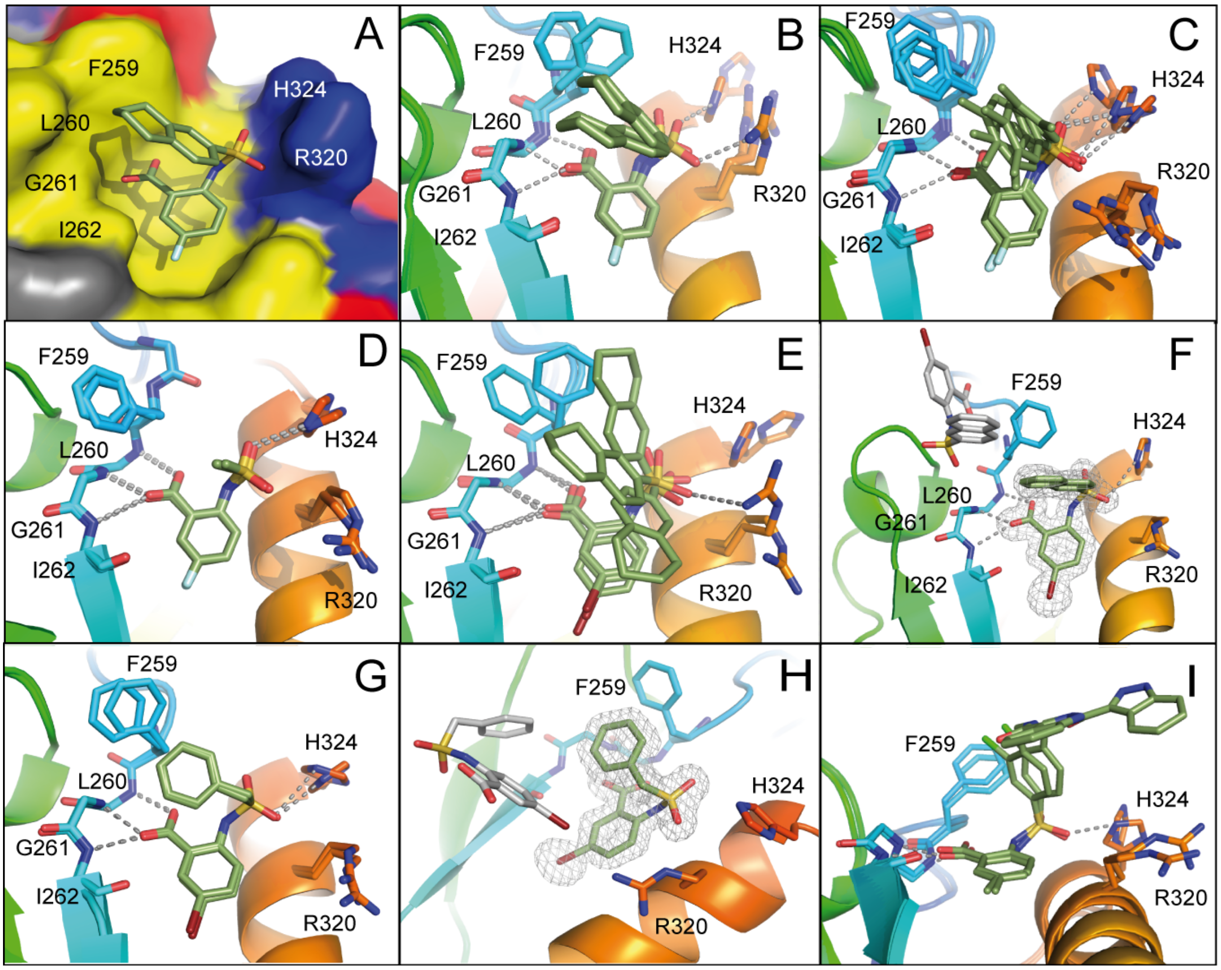

Figure S3: Detailed views of diverse compounds bound to the Dvl-3 PDZ domain. **A)** Surface representation of the Dvl-3 PDZ binding pocket with bound compound **3**. Positively charged amino acids are highlighted in blue and negatively charged amino acids in red. The hydrophobic Dvl-3 residues, contributing to compound binding, are colored yellow. **B-E), G) and I)** show detailed views of the binding pocket with bound compounds **3 (B)**, **5 (C), 6 (D), 7 (E), and 12 (G)**. Here, all Dvl-3 PDZ molecules per AU with their bound compounds are superimposed per species to demonstrate the binding variations per compound. Panels **F** and **H** present the additional unspecific compound binding to the Dvl-3 PDZ complex structures observed with compound **11 (F)** and compound **12 (H)**. Compound 18 (**I**) The non-specifically bound compounds are presented with grey sticks for covalent bonds to carbon atoms, and compounds bound to the canonical binding pocket of Dvl-3 PDZ domain are shown as green stick models enclosed in 2Fo-Fc electron density contoured at 1 sigma.

4. Cell viability assays of compounds 3, 7,8, 9, 10, (A) and 18, 20, 21 (B)

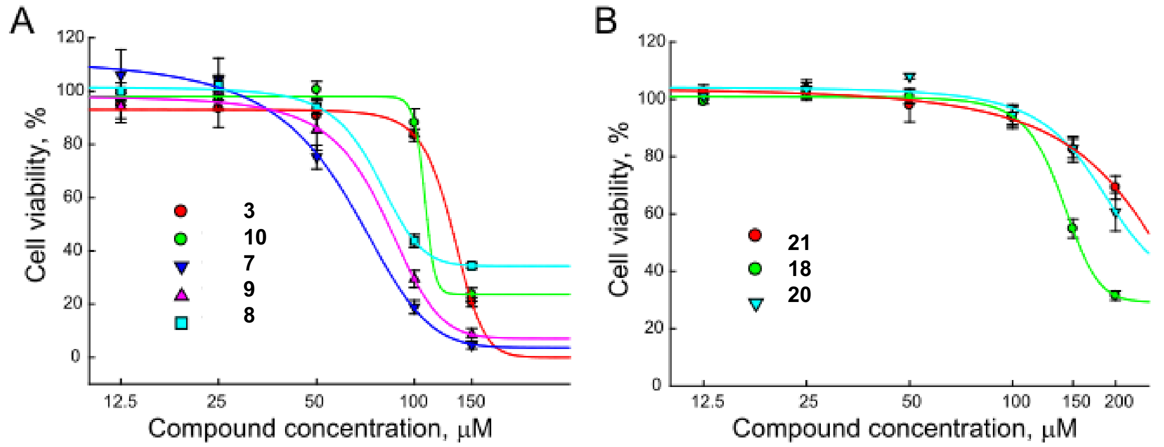

Figure S4: Cell viability assays of compounds 3, 7,8, 9, 10, (**A**) and 18, 20, 21 (**B**). Three independent biological replicas were performed in each case and error bars represent standard deviations.

5. ITC binding assays of compound 18 with Dvl-3 PDZ (A) and with Dvl-1 PDZ (B)

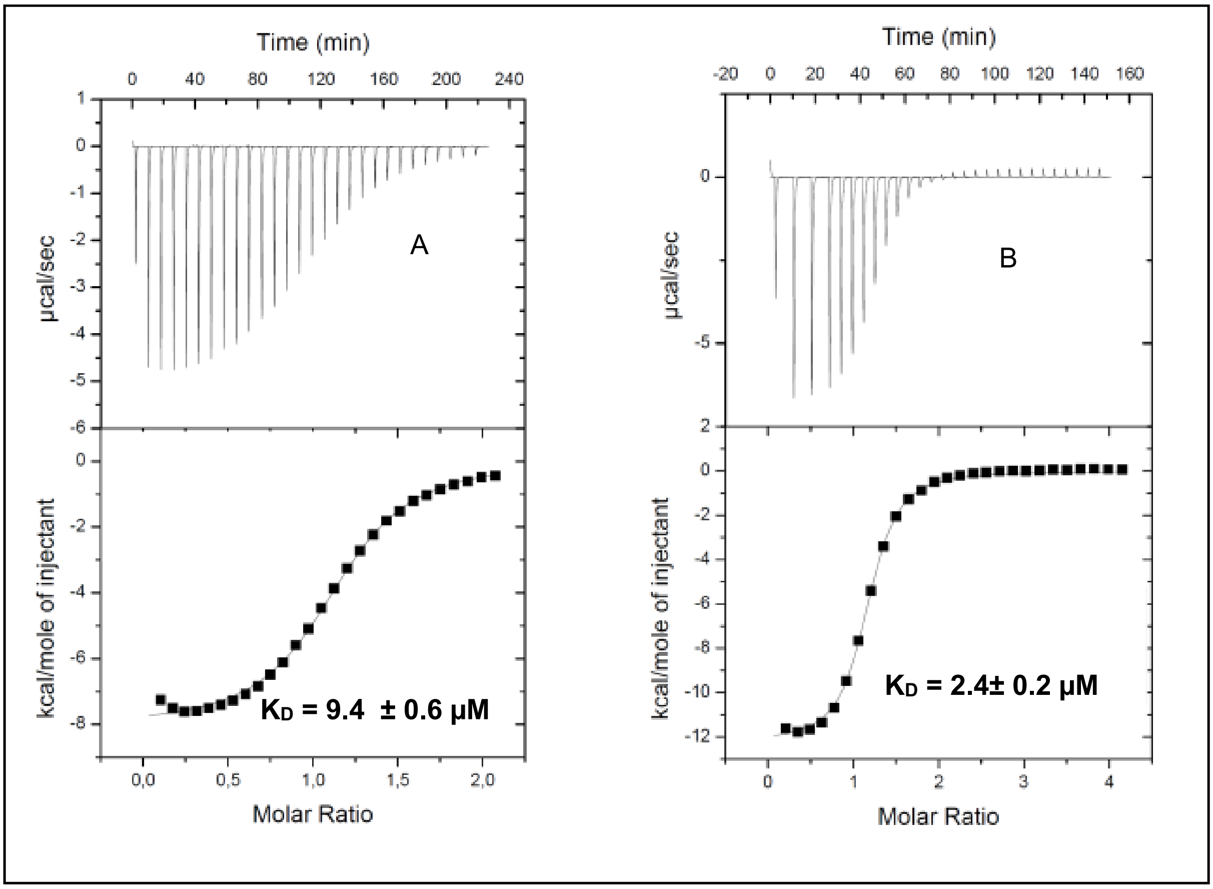

Figure S5: ITC binding assays of compound 18 with Dvl-3 PDZ (**A**) and with Dvl-1 PDZ (**B**). A 200 μM ligand solution containing 2% DMSO was injected 30 times in 10 μL aliquots at 120 s intervals with a stirring speed of 1000 rpm into a 1.4 mL sample cell containing the Dvl PDZ domain at a concentration of 20 μM and 2% DMSO. The data in A and B fitted to a one-site binding model with K_D determined by $1/K_A$ and $\Delta K_D = \Delta K_A/K_A^2$ and with n=1.14 and 1.12, respectively.

6. Structures of selected compounds used for comparison to our compounds

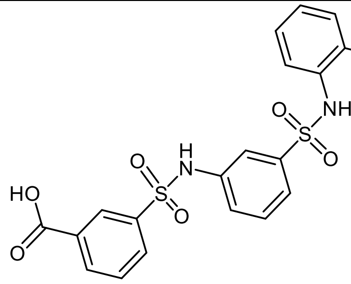 3-({3-[(2-carboxyphenyl)sulfamoyl]phenyl}sulfamoyl)benzoic acid (NPL-1011); (Hori et al., 2018)	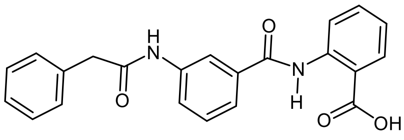 2-((3-(2-Phenylacetyl)amino)benzoyl)amino)benzoic acid, (CBC-322338/3289-8625); (Grandy et al., 2009, Hori et al., 2018)
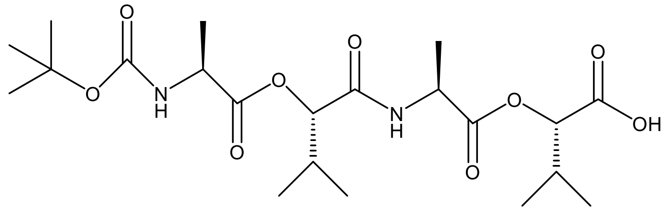 NSC668036; (Shan et al., 2005)	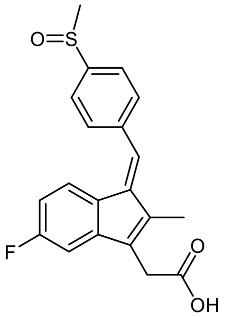 Sulindac (Lee et al., 2009a)
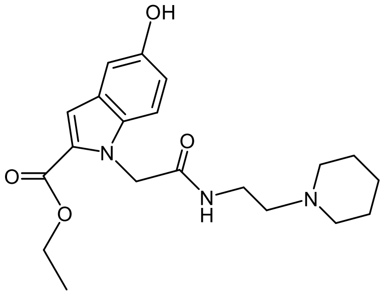 Ethyl 5-hydroxy-1-(2-oxo-2-((2-(piperidin-1-yl)ethyl)amino)ethyl)-1H-indole-2-carboxylate (KY-02327); (Kim et al., 2016)	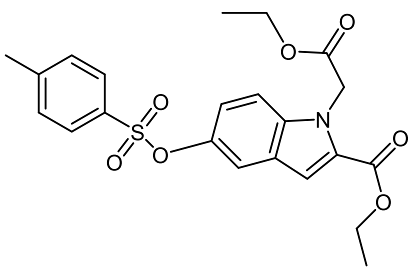 Ethyl 1-(2-ethoxy-2-oxoethyl)-5-(tosyloxy)-1H-indole-2-carboxylate (KY-02061); (Kim et al., 2016)
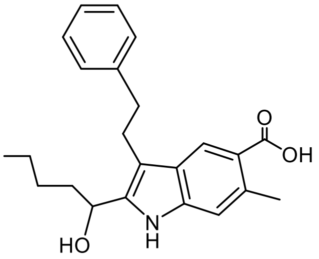 2-(1-Hydroxypentyl)-6-methyl-3-phenethyl-1H-indole-5-carboxylic acid (FJ9); (Fujii et al., 2007)	

Figure S6: Structures of selected compounds used for comparison to our compounds.

7. ITC data of selected compounds used for comparison to our compounds

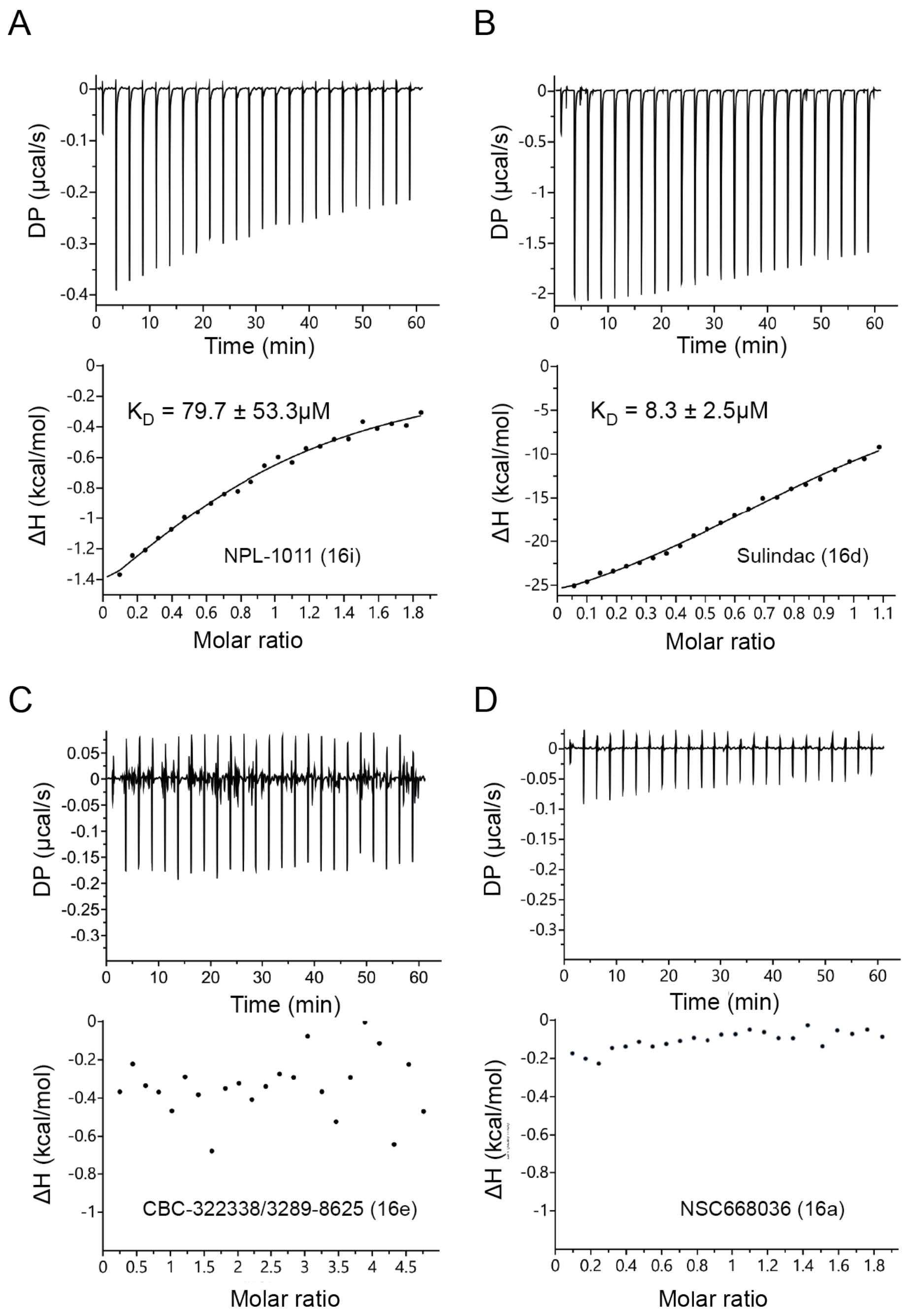

Figure S7: ITC data of A) NPL-1011(Hori et al., 2018), B) Sulindac (Lee et al., 2009a); C) CBC-322338/3289-8625 (Grandy et al., 2009, Hori et al., 2018) and D) NSC668036 (Shan et al., 2005), A) NPL-1011 revealed a binding of 79.7 ± 53.3 µM to DVL3-PDZ with N= 0.90 ± 0.08, ΔH = -2.7 ± 1.2 kcal/mol, ΔG -5.5 kcal/mol, -tΔS = -2.8 kcal/mol, whereas Sulindac shown in B) displayed an KD = 8.3 ± 2.5 µM with N=0.97 ±0.14, ΔH1 = -31.9 ± 5.3 kcal/mol, -tΔS1 = 24.9 kcal/mol. C) Compound CBC-322338/3289-8625 and D) NSC668036 did not show any binding to the Dvl-3-PDZ domain under the assay conditions applied.

8. NMR binding assay with 8-fold excess of reference compound 3289-8625

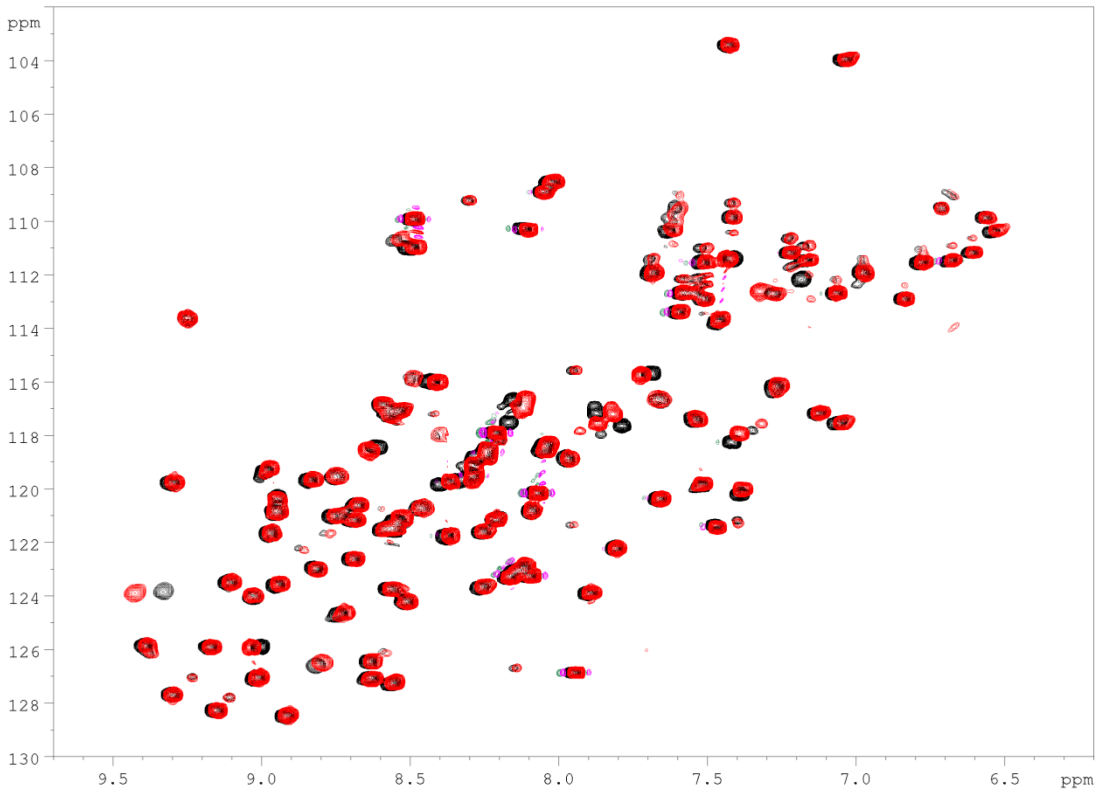

Figure S8: ^{1}H-^{15}N HSQC spectra of Dvl-3 PDZ domain alone (black, concentration of 50 µM) and in the presence of eight-fold excess of compound 3289-8625. For a comparison of effect strength see Figure S2 (8-fold excess is the maximum ligand concentration used there), Table S1 and Table 1.

9. Purity check of compounds

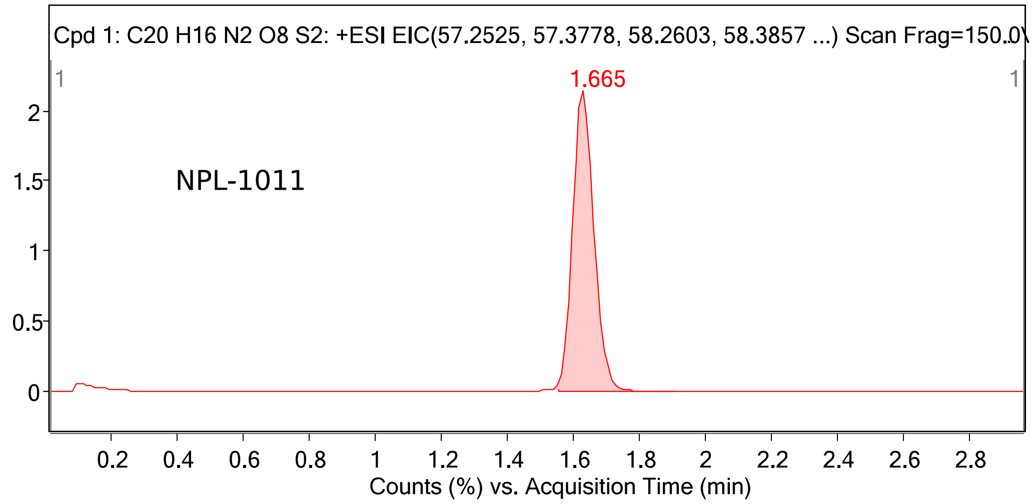

MS Spectrum

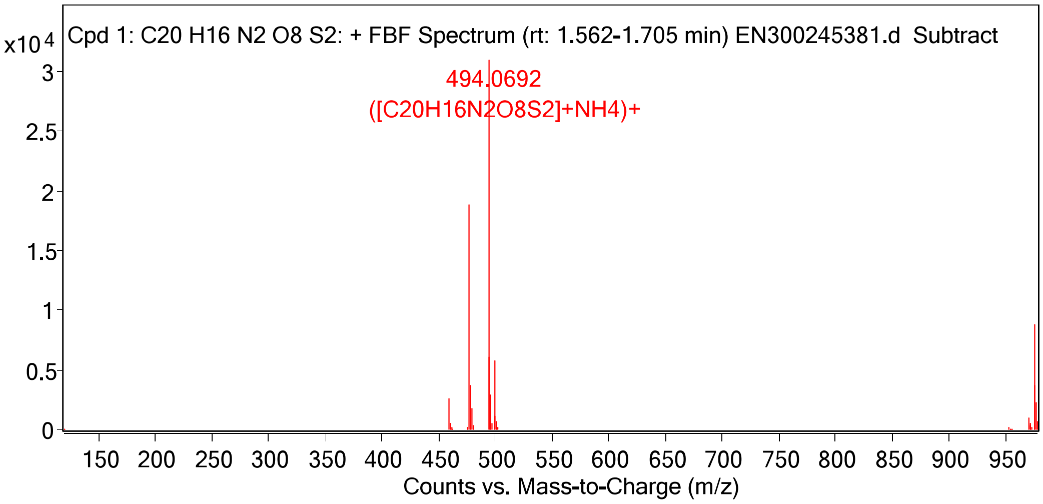

MS Zoomed Spectrum

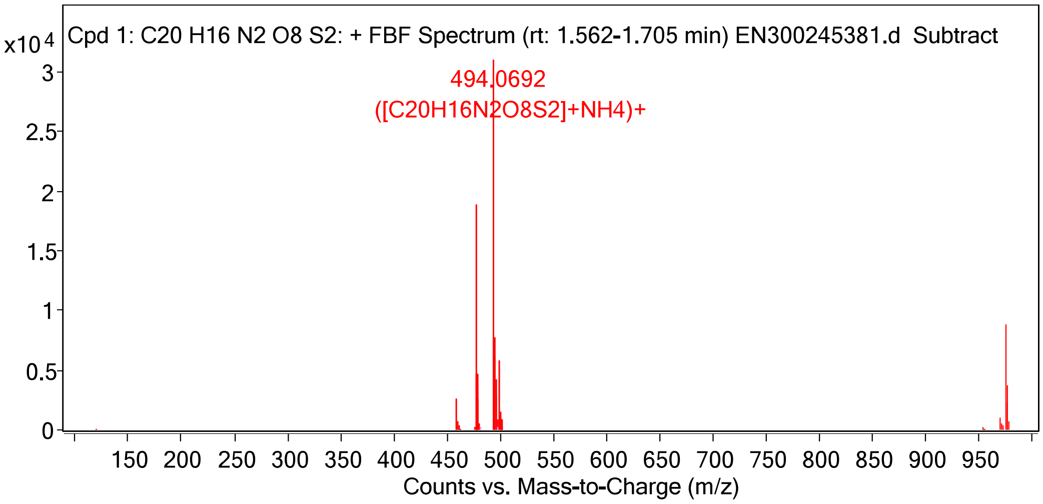

Figure S9a: Purity check of NPL-1011 compound

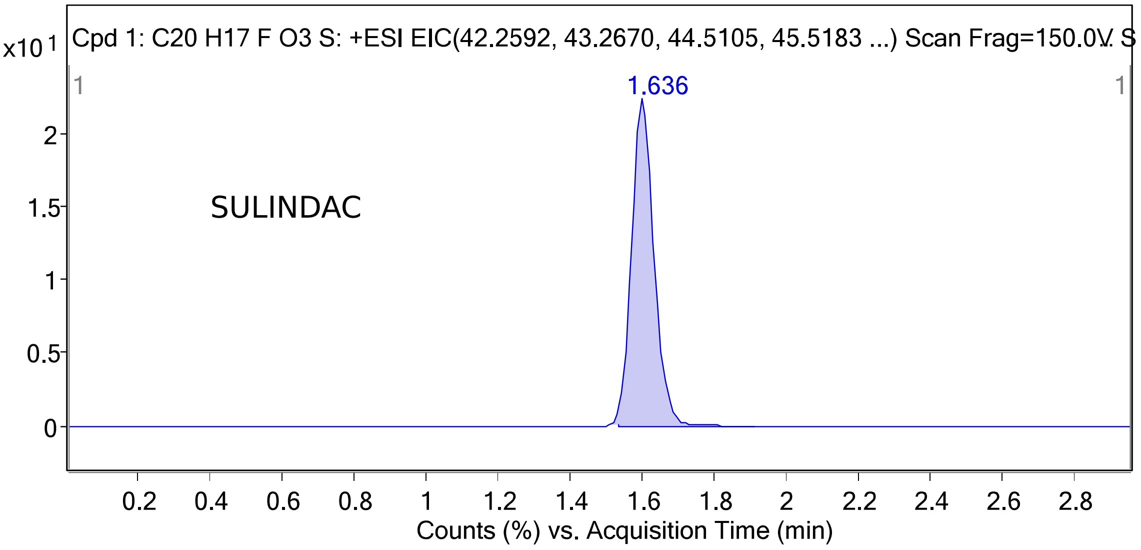

MS Spectrum

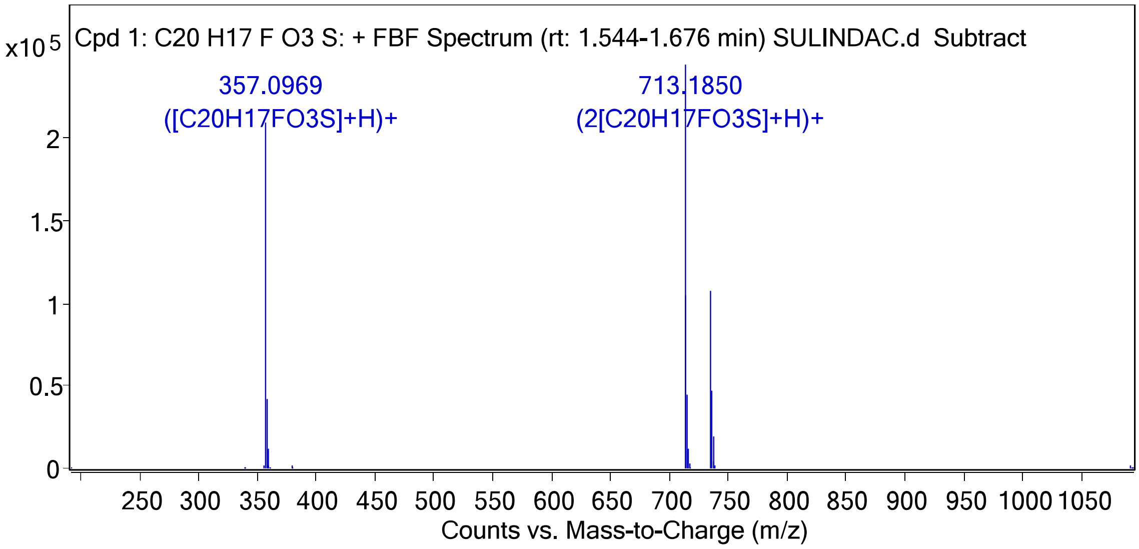

MS Zoomed Spectrum

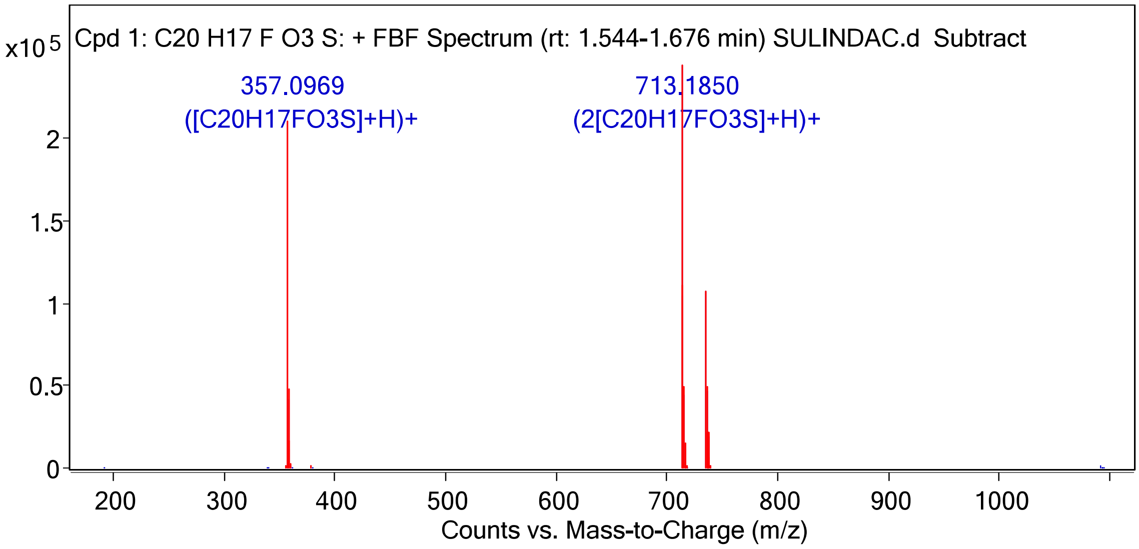

Figure S9b: Purity check of Sulindac compound

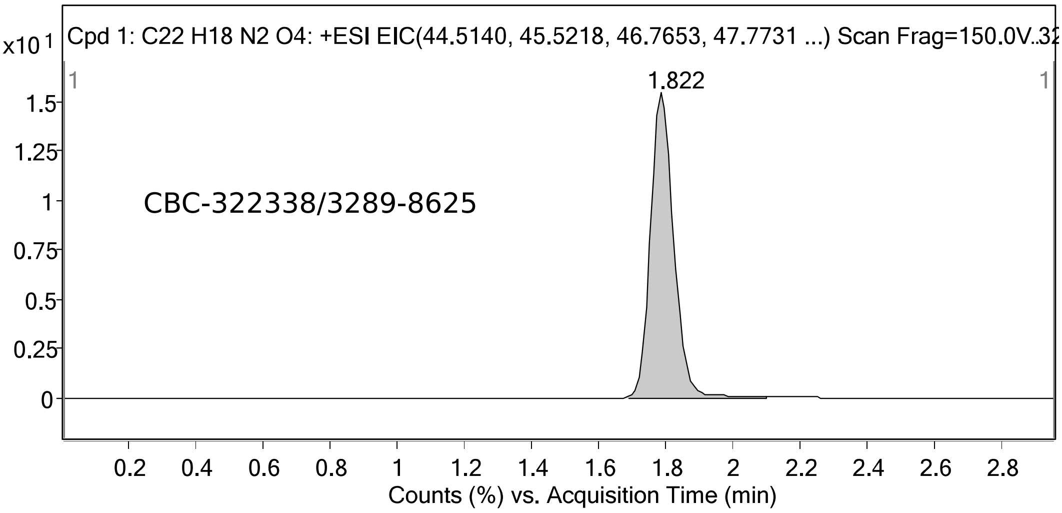

MS Spectrum

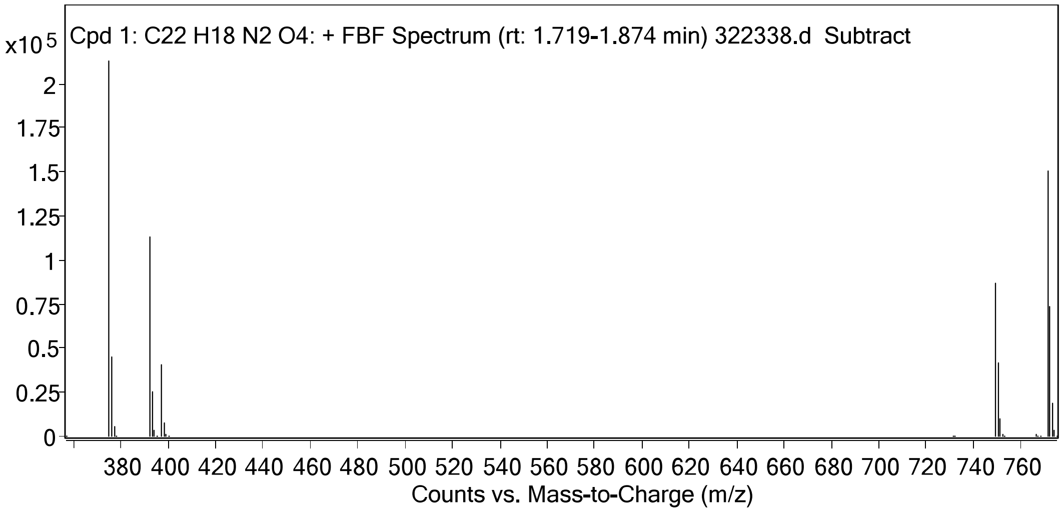

MS Zoomed Spectrum

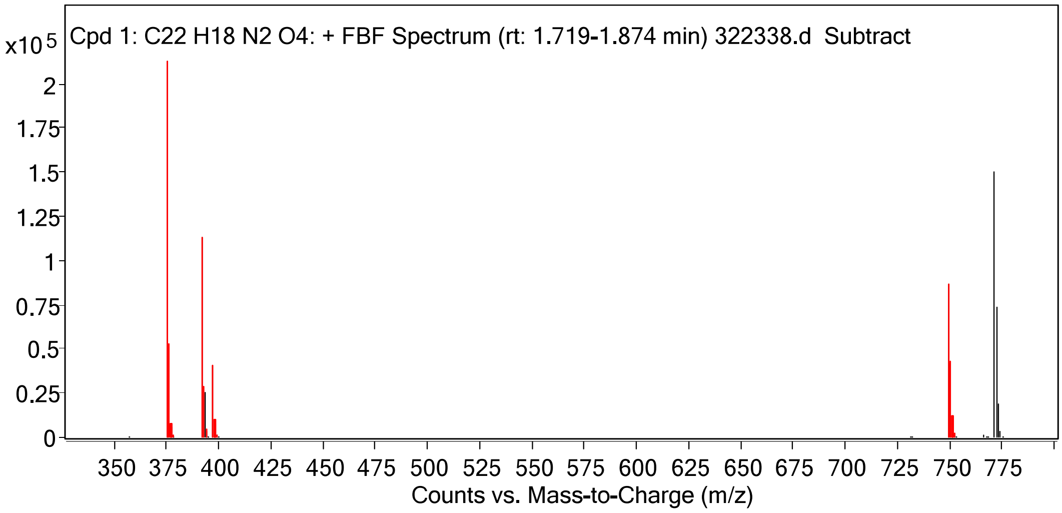

Figure S9c: Purity check of CBC-322338/3289-8625 compound

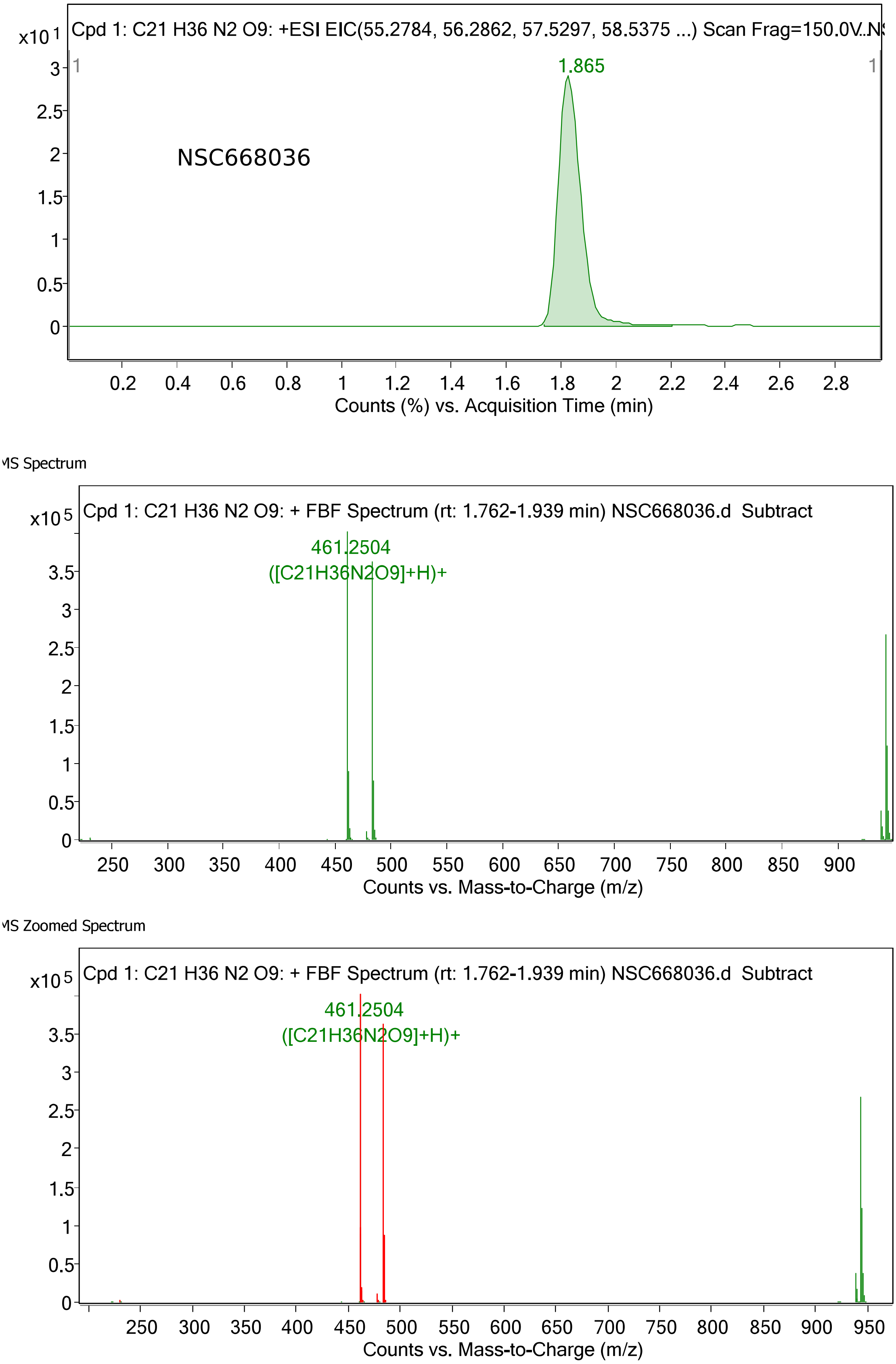

Figure S9d: Purity check of NSC668036 compound

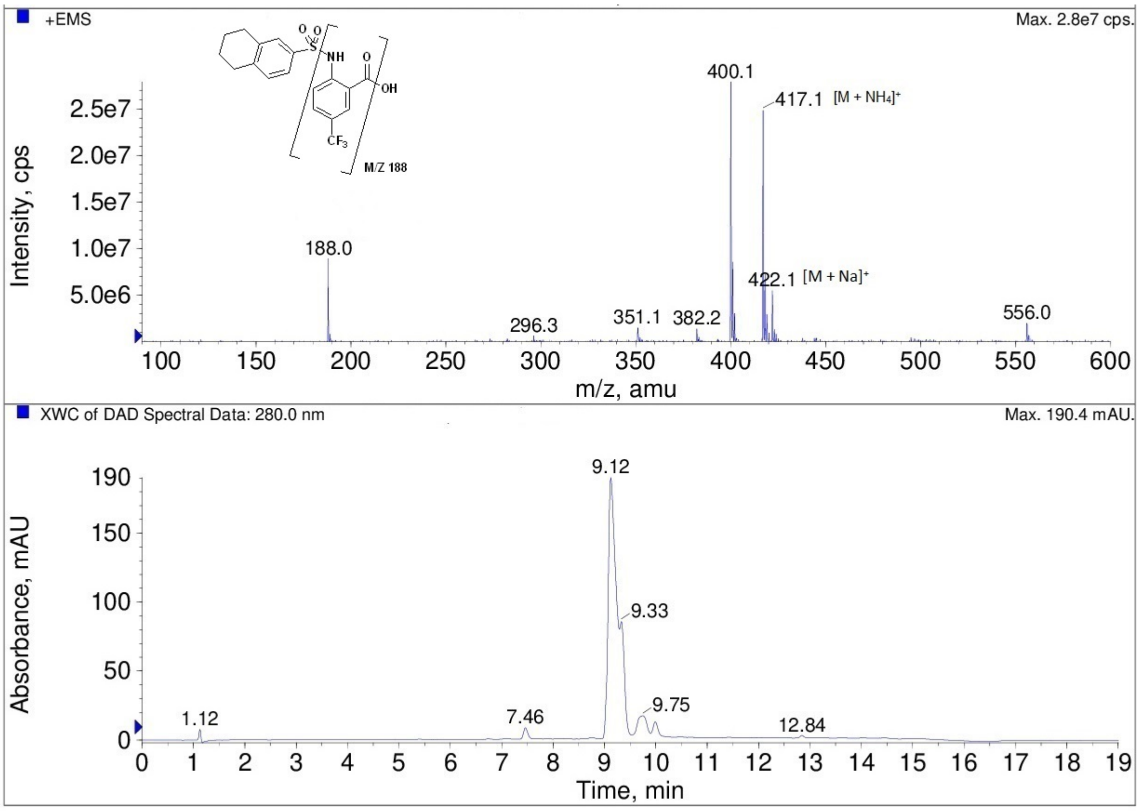

Figure S9e: LCMS of intermediate compound 8 : Peak at 1.1 refer to the instrumental signal prior to sample injection

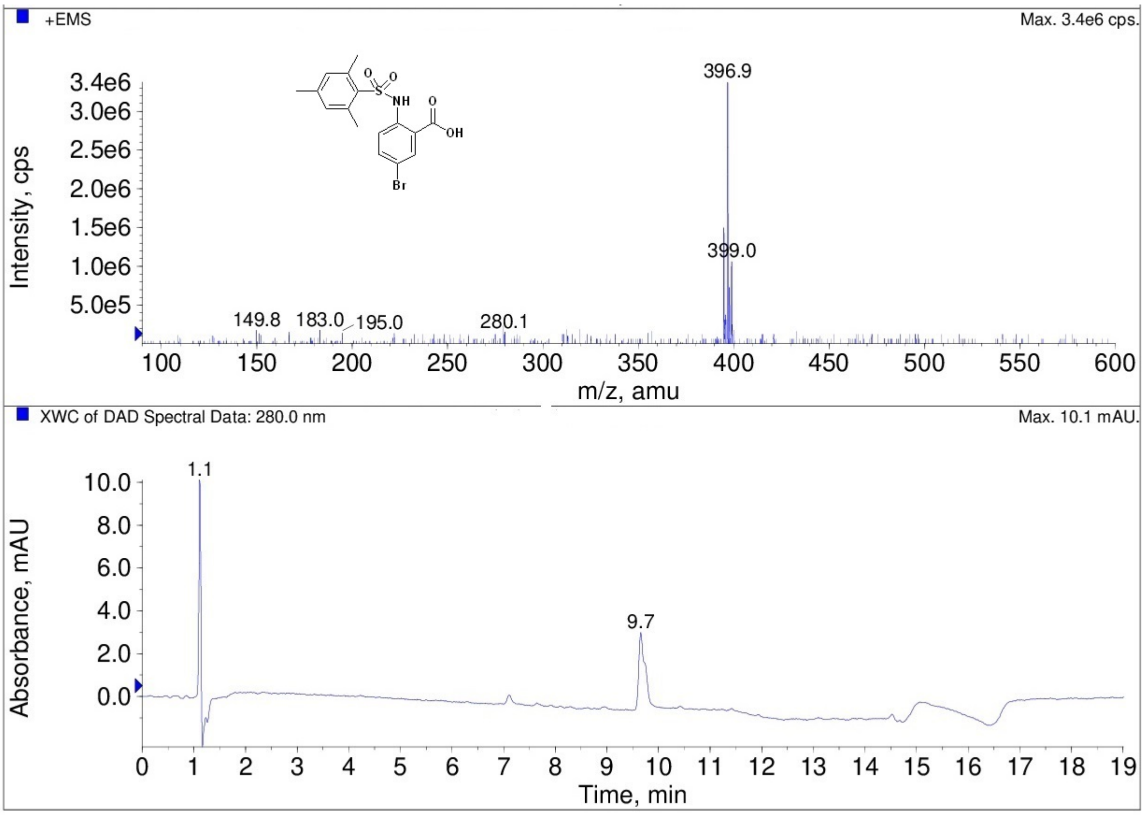

Figure S9f: LCMS of intermediate compound 14 : Peak at 1.1 refer to the instrumental signal prior to sample injection

10. Chemical shift perturbation values of Dvl-3 PDZ and Dvl-1 PDZ for compounds (2-21)

	ID	R_1	R_2	ΔCSP(ppm) Dvl-3PDZ	ΔCSP(ppm) Dvl-1 PDZ
	2	F	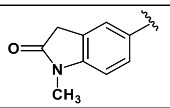	0.18	0.2
	3	F	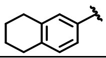	0.27	0.086
	4	F	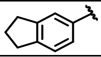	0.26	0.3
	5	F	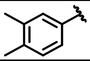	0.23	0.15
	6	F	CH_3	0.11	
	7	Br	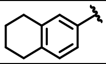	0.23	0.3
	8	CF_3	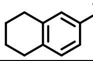	0.38	0.26
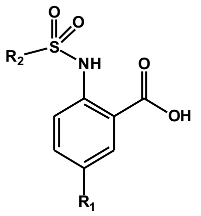	9	Cl	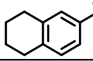	0.28	0.34
	10	CH_3	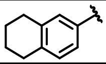	0.26	0.31
	11	Br	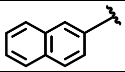	0.31	0.18
	12	Br	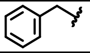	0.21	0.29
	13	Br	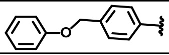	0.2	0.22
	14	Br	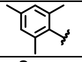	0.31	0.26
	15	CF_3	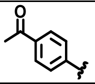	0.28	0.24
	16	CF_3	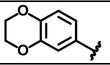	0.36	0.08
	17	CF_3	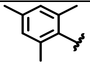	0.21	0.23
	18	CH_3	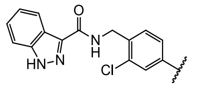	0.30	0.36
	19	CH_3	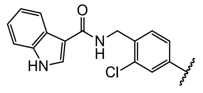	0.36	0.32
	20	CH_3	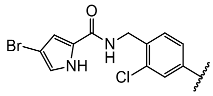	0.35	0.36
	21	CH_3	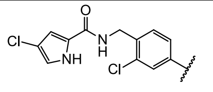	0.34	0.34

Table S1: Chemical shift perturbation values of Dvl-3 PDZ and Dvl-1 PDZ for compounds (2 – 21). ΔCSP is the mean value of 3 amino acid residues showing strong chemical shift perturbations.

11. Data collection and refinement statistics of compounds 3, 5, 6, 7

**Dvl3 with compound**	**3**	**5**	**6**	**7**
**Data collection**				
Space group	I4	$P2_12_12_1$	$P6_1$	I4
a, *b*, *c* (Å)	76.3, 76.3, 72.4	56.8, 70.0, 87.2	87.3, 87.3, 57.8	76.3, 76.3, 72.6
α, β, γ (°)	90.0, 90.0, 90.0	90.0, 90.0, 90.0	90.0, 90.0, 120.0	90.0, 90.0, 90.0
Resolution (Å)*	30.0-1.43 (1.47-1.43)	34.6-1.60 (1.64-1.60)	34.8-1.67 (1.71-1.67)	30.9-1.85 (1.90-1.85)
R_{meas}*	4.4 (57.9)	3.8 (80.0)	5.5 (77.4)	5.8 (105.0)
$< I / \sigma(I) >$*	22.1 (3.2)	23.6 (2.3)	19.1 (2.5)	20.5 (2.1)
Completeness (%)*	100 (100)	99.7(99.8)	99.9 (100)	99.8 (99.6)
Redundancy*	5.4 (5.3)	4.8 (4.8)	5.7 (5.7)	7.4 (7.3)
**Refinement**				
No. total reflections	207003 (15053)	223464 (16344)	165069 (12220)	133118 (9391)
No. unique reflections	38358 (2826)	46555 (3405)	29202 (2161)	17796 (1282)
R_{work} / R_{free}	0.160 / 0.204	0.199/0.249	0.179/0.218	0.197/0.246
Mean B factor (Å^2)	16.1	24.3	21.4	20.6
Bond lengths (Å)	0.016	0.017	0.018	0.018
Bond angles (º)	1.867	1.753	1.762	1.805
Molecules in AU	2	4	2	2
**Ramachandran**				
Favoured region (%)	97.0	98.0	96.6	96.4
Outlier region (%)	0	0.3	0	0

* Data in highest resolution shell are indicated in parenthesis.

Table S2: Data collection and refinement statistics.

12. Data collection and refinement statistics of compounds 11, 12, 18

**Dvl3 with compound**	**11**	**12**	**18**
**Data collection**			
Space group	I422	$P6_1$	$P6_422$
a, *b*, *c* (Å)	78.6, 78.6, 77.8	85.3, 85.3, 58.9	89.3, 89.3, 131.6
α, β, γ (°)	90.0, 90.0, 90.0	90.0, 90.0, 120.0	90.0, 90.0, 120.0
Resolution (Å)*	32.0-1.58 (1.62-1.58)	34.6-1.48 (1.52-1.48)	34.8-2.76 (2.83-2.76)
R_{meas}*	6.4 (69.0)	6.7 (80.5)	14.2 (82.6)
$< I / \sigma(I) >$*	18.1 (2.9)	18.4 (3.2)	21.4 (4.1)
Completeness (%)*	99.5 (100)	100 (100)	99.9 (100)
Redundancy*	7.1 (7.2)	8.0 (8.0)	12.6 (13.3)
**Refinement**			
No. total reflections	120373.4 (8848.8)	326040 (24096)	107037 (8073)
No. unique reflections	16954 (1229)	40755 (3012)	8495 (607)
R_{work} / R_{free}	0.182 / 0.221	0.148/0.178	0.242/0.299
Mean B factor ($Å^2$)	23.0	22.7	36.6
Bond lengths (Å)	0.021	0.019	0.013
Bond angles (º)	2.028	1.933	1.442
Molecules in AU	1	2	2
Favoured region (%)	98.0	97.8	98.0
Outlier region (%)	0	0.0	0

* Data in highest resolution shell are indicated in parenthesis.

Table S3: Data collection and refinement statistics.

13. Selectivity of ligands derived from chemical shift perturbation of compounds tested at other PDZ domains

CP Id	PDZ							
	Dvl-1	Dvl-3	PSD95-1	PSD95-2	PSD95-3-	Shank-3	a-1-Syn	AF-6
18	0.32	0.30	0.05	0.1	0.05	0.01	0.08	0.01
20	0.3	0.36	0.06	0.09	0.06	0.05	0.07	0.01
21	0.3	0.36	0.07	0.09	0.1	0.05	0.08	0.01

Table S4: Selectivity of ligands derived from chemical shift perturbation of compounds tested at other PDZ domains. The PDZ domain set includes PSD95-1, PSD95-2, PSD95-3, Shank-3, α-1 Syn and AF-6.ΔCSP is the mean value of 3 amino-acid residues showing chemical shift perturbation

14. Details of Multifilter routines

PDB structure ID	Distance from a ligand atom to PDZ atom			H-bond threshold	Resulting number of compounds
	2.5 Å		4 Å		
2os6, model 8	Gly21 HN	Leu22 HN	Leu22 CD1	3	228
2dlu, model 1	Gly29 HN	Phe30 HN	Phe30 CE1	4	204
2o2t, chain B	Gly149 HN	Phe150 HN	Phe150 CE1	4	332
1va8, model 3	Gly40 HN	Ala41 HN	Leu93 CG	4	284
1uhp, model 8	Gly22 HN	Phe23 HN	Phe86 CD2	3	329
3lnx, chain A	Leu18 HN	Gly19 HN	Ile20 CG1	4	220

Table S5: Details of Multifilter routines.

15. Smiles codes and Compounds ID

STRUCTURE / ID in paper	MOLECULE	MW	COMPANY ID	SMILES CODE
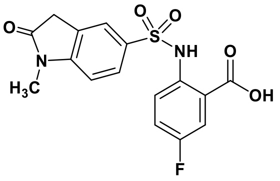 2	$C_{16}H_{13}FN_2O_5S$	364,3	ENAMINE T58 630 40	CN1C(=O)CC2=C1C=CC(=C2)S(=O)(=O)NC3=C(C=C(C=C3)F)C(=O)O
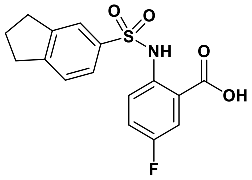 3	$C_{17}H_{16}FNO_4S$	349.4	ENAMINE T6324911	O=C(O)c1cc(F)ccc1NS(=O)(=O)c3ccc2CCCCc2c3
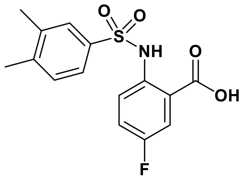 4	$C_{16}H_{14}FNO_4S$	335.4	ENAMINE T6324915	O=C(O)c1cc(F)ccc1NS(=O)(=O)c3ccc2CCCc2c3
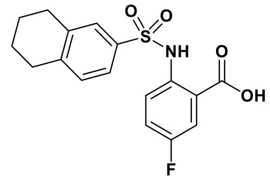 5	$C_{17}H_{16}FNO_4S$	349.4	ENAMINE T6305470	C1CCC2=C(C1)C=CC(=C2)S(=O)(=O)NC3=C(C=C(C=C3)F)C(=O)O
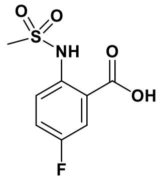 6	$C_8H_8FNO_4S$	233.22	FMP	CS(=O)(=O)Nc1ccc(F)cc1C(=O)O
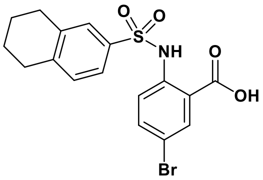 7	$C_{17}H_{16}BrNO_4S$	410.3	ENMINE 28744264	O=C(O)c1cc(Br)ccc1NS(=O)(=O)c3ccc2CCCCc2c3
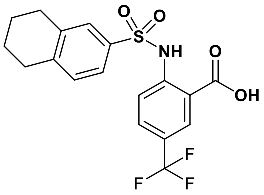 8	$C_{18}H_{16}F_3NO_4S$	399,383	FMP	O=C(O)c1cc(C(F)(F)F)ccc1NS(=O)(=O)c3ccc2CCCCc2c3
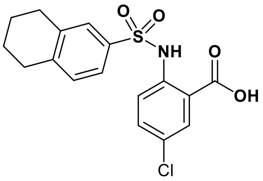 9	$C_{17}H_{16}ClNO_4S$	365.8	ENAMINE 28775339	O=C(O)c1cc(Cl)ccc1NS(=O)(=O)c3ccc2CCCCc2c3

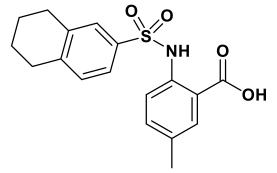 **10**	$C_{18}H_{19}NO_4S$	345.4	ENAMINE 233895416	Cc3ccc(NS(=O)(=O)c2ccc1CCCCc1c2)c(C(=O)O)c3
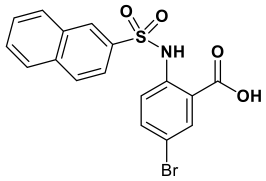 **11**	$C_{17}H_{12}BrNO_4S$	406.3	FMP	O=C(O)c1cc(Br)ccc1NS(=O)(=O)c3ccc2ccccc2c3
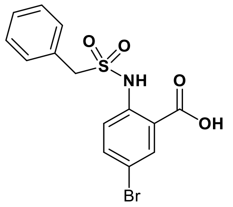 **12**	$C_{14}H_{12}BrNO_4S$	370.22	FMP	O=C(O)c1cc(Br)ccc1NS(=O)(=O)Cc2ccccc2
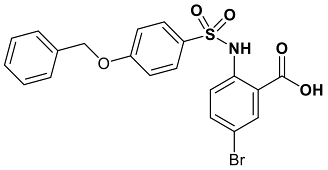 **13**	$C_{20}H_{16}BrNO_5S$	462.314 1	FMP	O=C(O)c1cc(Br)ccc1NS(=O)(=O)c3ccc(COc2ccccc2)cc3
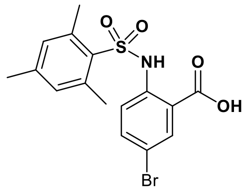 **14**	$C_{16}H_{16}BrNO_4S$	398.3	FMP	Cc2cc(C)c(S(=O)(=O)Nc1ccc(Br)cc1C(=O)O)c(C)c2
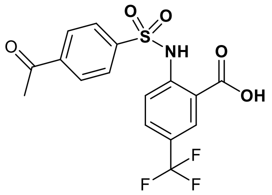 **15**	$C_{16}H_{12}F_3NO_5S$	387.329 7	FMP	CC(=O)c2ccc(S(=O)(=O)Nc1ccc(C(F)(F)F)cc1C(=O)O)cc2
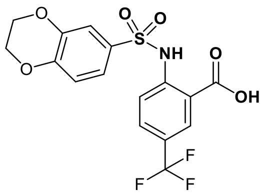 **16**	$C_{16}H_{12}F_3NO_6S$	403,329	FMP	O=C(O)c1cc(C(F)(F)F)ccc1NS(=O)(=O)c3ccc2OCCOc2c3
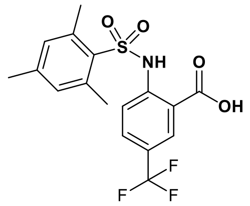 **17**	$C_{17}H_{16}F_3NO_4S$	387,372	FMP	Cc2cc(C)c(S(=O)(=O)Nc1ccc(C(F)(F)F)cc1C(=O)O)c(C)c2

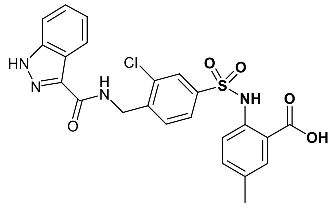 **18**	$C_{23}H_{19}ClN_4O_5S$	498,939	ENAMINE Z1098340488	Cc4ccc(NS(=O)(=O)c3ccc(CNC(=O)c1n[nH]c2ccccc12)c(Cl)c3)c(C(=O)O)c4
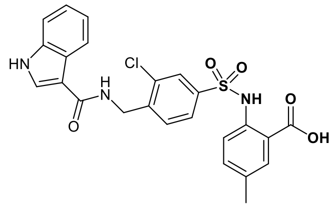 **19**	$C_{24}H_{20}ClN_3O_5S$	497,952	ENAMINE Z1098340555	Cc4ccc(NS(=O)(=O)c3ccc(CNC(=O)c1c[nH]c2ccccc12)c(Cl)c3)c(C(=O)O)c4
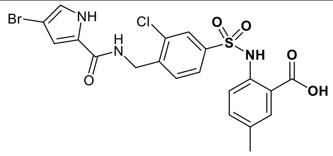 **20**	$C_{20}H_{17}BrClN_3O_5S$	526,788	ENAMINE Z1098340559	Cc3ccc(NS(=O)(=O)c2ccc(CNC(=O)c1cc(Br)c[nH]1)c(Cl)c2)c(C(=O)O)c3
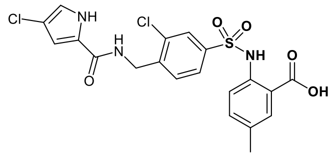 **21**	$C_{20}H_{17}Cl_2N_3O_5S$	482,337	ENAMINE Z1098340560	Cc3ccc(NS(=O)(=O)c2ccc(CNC(=O)c1cc(Cl)c[nH]1)c(Cl)c2)c(C(=O)O)c3
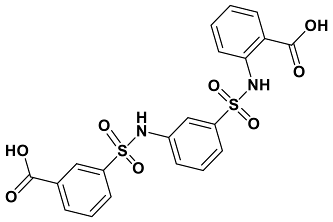 **NPL-1011**	$C_{20}H_{16}N_2O_8S_2$	476.5	ENAMINE EN300 -245381	C1=CC=C(C(=C1)C(=O)O)NS(=O)(=O)C2=CC=CC(=C2)NS(=O)(=O)C3=CC=CC(=C3)C(=O)O
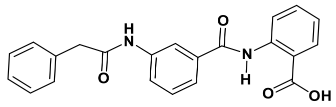 CBC-322338/3289-8625 (Grandy et al., 2009, Hori et al., 2018)	$C_{22}H_{18}N_2O_4$	374.4	MERCK 322338-10MG	C1=CC=C(C=C1)CC(=O)NC2=CC=CC(=C2)C(=O)NC3=CC=CC=C3C(=O)O
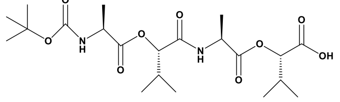 NSC668036 (Shan et al., 2005)	$C_{21}H_{36}N_2O_9$	460.5	SIGMA SML0046	CC(C)C(C(=O)NC(C)C(=O)OC(C(C)C)C(=O)O)OC(=O)C(C)NC(=O)OC(C)(C)C
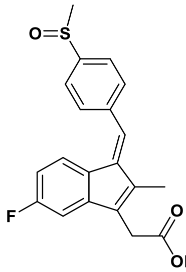 Sulindac (Lee et al., 2009a)	$C_{20}H_{17}FO_3S$	356.4	SIGMA S8139-5G	CC1=C(C2=C(C1=CC3=CC=C(C=C3)S(=O)C)C=CC(=C2)F)CC(=O)O

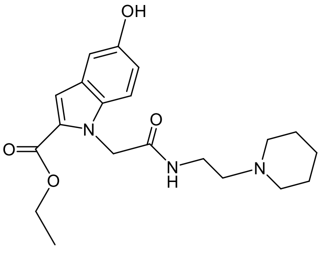 (KY-02327) (Kim et al., 2016)	$C_{20}H_{27}N_3O_4$	373.4	D&C Chemicals<br><br>DC21213	CCOC(=O)C1=CC2=C(N1CC(=O)NCCN3CCCCC3)C=CC(=C2)O
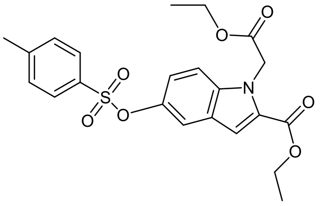 (KY-02061) (Kim et al., 2016)	$C_{22}H_{23}NO_7S$	445.5	D&C Chemicals<br><br>**DC21213**	CCOC(=O)CN1C2=C(C=C(C=C2)OS(=O)(=O)C3=CC=C(C=C3)C)C=C1C(=O)OCC
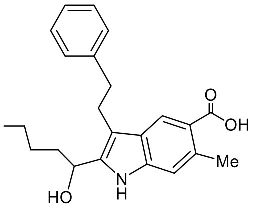 (FJ9) (Fujii et al., 2007)	$C_{23}H_{27}NO_3$	365.5	Toronto Research Chemicals<br><br>**H939980**	CCCCC(C1=C(C2=C(N1)C=C(C(=C2)C(=O)O)C)CCC3=CC=CC=C3)O

Table S6: Smiles codes and Compounds ID. Compounds containing literature indication are those used for comparison to our compounds.

16: NMR characterization of synthesized compounds (8 , 11 , 13 , 14 , 15 , 16 , 17)

2-(5,6,7,8-tetrahydronaphthalene-2-sulfonamido)- 5- (trifluoromethyl) benzoic acid (8)

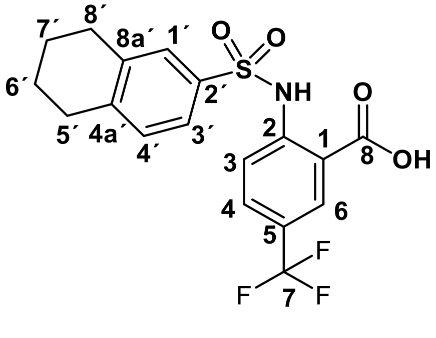

$C_{18}H_{16}F_3NO_4S$
M=399,4 g/mol

(0.52 g, 74% yield) **^{1}H-NMR** (300 MHz, DMSO-d6): δ = 11.77 [s, 1H, COOH], 8.13 [s, 1H, NH], 7.85 [d, $^4J_{6,4}$ = 2.1 Hz, 1H, 6-H_{Ar}] 7.62 [d, $^4J_{1',3'}$ = 2.1Hz , 1H , 1´-H_{Ar}] 7.53 [dd, $^3J_{4,3}$ = 7.1 Hz, $^4J_{4,6}$ = 2.1 Hz, 4-H_{Ar}] 7.36 [dd, $^3J_{3'4'}$ = 7.5 Hz, $^4J_{3',1'}$ = 2.4 Hz, 1H, 3´-H_{Ar}] 7.15 [d, $^3J_{4',3'}$ = 7.5Hz, 1H,4´-H_{Ar}] ,

6.90 [d, $^{3}J_{3,4}$ = 7.1Hz, 1H , 3-H_{Ar}] 2.73 (m, 4H, CH_2); 1.6 (m, 4H, CH_2). **^{13}C-NMR** (75 MHz, DMSO-d6): δ = 169.1(C, C_{Ar}-8], 152.7(C, C_{Ar}-2), 143.8 (C, C_{Ar}-4a′), 138.7(C, C_{Ar}-2′), 135.9 (C, C_{Ar}-8a′), 130.4(CH, C_{Ar}-4), 128.7 (CH, C_{Ar}-6), 127.5 (CH, C_{Ar}-1′), 124.0 (CH, C_{Ar}-4′), 121.6 (C, C-6), 118.2 (C, C_{Ar}-5), 116.9 (C, C_{Ar}-3), 29.0 (CH_2, C-8′), 28.8 (CH_2, C-5′), 22.3 (CH_2, C-6′), 22.2 (CH_2, C-7′); mp: 177°C; MS (ESI) *m*/z:calcd. for $C_{18}H_{16}F_3NO_4S$, 399; found, 400 $[M+H]^+$.

5-bromo-2-(naphthalene-2-sulfonamido) benzoic acid (11)

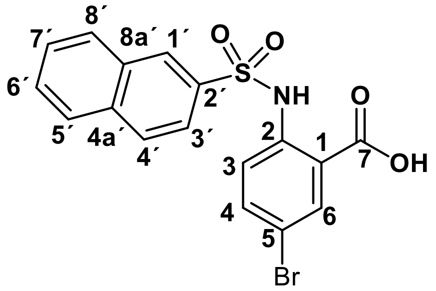

$C_{17}H_{12}BrNO_4S$
M = 406.3g/mol

(0.13 g, 67% yield) **^{1}H-NMR** (300 MHz, DMSO-d6): δ = 10.2 [s, 1H, COOH], 9.8 [s, 1H, NH] 8.59 [d, $^{4}J_{1',3'}$ = 1.4 Hz, 1 H, 1′-H_{Ar}], 8.17 [d, $^{3}J_{8',7'}$ = 7.8 Hz, 1 H, 8′-H_{Ar}], 8.10 [d, $^{3}J_{4'3'}$ = 8.8 Hz, 1H, 4′-H_{Ar}], 8.02 [d, $^{3}J_{5',6'}$ = 7.8 Hz, 1H, 5′-H_{Ar}], 7.93 [d, $^{4}J_{6,4}$ = 2.4 Hz, 1H, 6-H_{Ar}], 7.77 [dd, $^{3}J_{3',4'}$ = 8.8 Hz, $^{4}J_{3',1'}$ = 1.4Hz ,1 H, 3′-H_{Ar}], 7.72 – 7.65 [m, 3H, 4-H_{Ar}, 6′-H_{Ar}, 7′-H_{Ar}] , 7.51 [d, $^{3}J_{3,4}$ = 8.9 Hz, 1H, 3-H_{Ar}]. **^{13}C-NMR** (75 MHz, DMSO-d6): δ = 168.2 (C, C-7) ,138.8 (C, C_{Ar}-2),136.8 (CH, C_{Ar}-4),135.3 (C, C_{Ar}-4a′),134.4 (C, C_{Ar}-8a′) ,133.4 (CH, C_{Ar}-6) ,131.4 (CH, C_{Ar}-6′), 129.3 (CH, C_{Ar}-4′) ,128.5 (CH, C_{Ar}-8′),127.8 (2xCH, C_{Ar}-5′, C_{Ar}-7′) 121.6 (CH, C_{Ar}-3′),120.6 (CH, C_{Ar}-3),119.0(C, C_{Ar}-1) ,114.9 (C, C_{Ar}-5). Mp: 199°C; (ESI) *m*/z: calcd.for $C_{17}H_{11}BrNO_4S^-$, 403.9560; found, 403.9613 $[M-H]^-$.

5-bromo-2-(phenylmethylsulfonamido)benzoic acid (12)

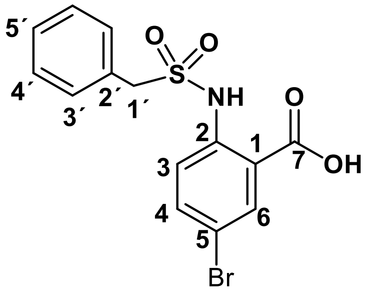

$C_{14}H_{12}BrNO_4S$
M=370.2 g/mol

(0.07g, 42% yield) **^{1}H-NMR** (300 MHz, DMSO-d6): δ = 10.57 [s, 1H, COOH], 8.05 [d, $^4J_{6,4}$ = 2.4 Hz, 1 H, 6-H_{Ar}], 7.75 [dd, $^3J_{4,3}$ = 8.9 Hz, $^4J_{4,6}$ = 2.4Hz ,1H, H-4$_{Ar}$], 7.49 [d,$^3J_{3,4}$ = 8.9 Hz,1H, 3-H_{Ar}], 7.33 – 7.28 [m, 3H, 3´-H_{Ar} , 5´-H_{Ar}], 7.23 – 7.20 [m, 2H, 4´-H_{Ar}], 5.75 [s, 1H, NH], 4.72 [s, 2H, 1´-H]. **^{13}C-NMR** (75 MHz, DMSO-d6): δ = 168.3 (C, C-7) , 139.9 (C, C_{Ar}-2), 137(CH, C_{Ar}-4), 133.4 (CH, C_{Ar}-6), 130.7 (CH, C_{Ar}-3´) , 128.6 (C, C_{Ar}-2´) , 128.4 (CH, C_{Ar}-5´) , 128.3 (CH, C_{Ar}-4´) , 119.5 (CH, C_{Ar}-3) , 117.5 (C, C_{Ar}-1) , 113.9 (C, C_{Ar}-5) , 57.4 (CH_2, C-1´). Mp: 216°C; (ESI) *m*/z: calcd.for $C_{14}H_{11}BrNO_4S^-$,367.9860; found , 367.9878 $[M-H]^-$.

5-bromo-2-(4-(phenoxymethyl)phenylsulfonamido)benzoic acid (13)

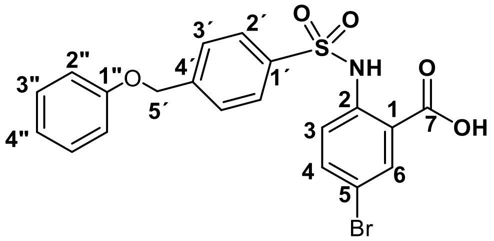

$C_{20}H_{16}BrNO_5S$
M=462.3 g/mol

(0.6 g, 29% yield) 1H-NMR (300 MHz, DMSO-d6): δ = 7.97 [d, $^4J_{6,4}$ = 2.4 Hz, 1H, 6-H_{Ar}), 7.85 (d, $^3J_{2´,3´}$ = 8.3 Hz, 2H, 3´-H_{Ar}), 7.73 [dd, $^3J_{4,3}$ = 8.9 Hz, $^4J_{4,6}$ = 2.4Hz , 1H,4-H_{Ar}], 7.63 [d, $^3J_{2´,3}$´ = 8.3 Hz, 2H, 2´-H_{Ar}], 7.47 [d, $^3J_{3,4}$ = 8.9 Hz, 1H, 3-H_{Ar}], 7.29 [dd, $^3J_{3",2"}$ = $^3J_{3",4"}$ =7.3 Hz, 2H, 3"-H_{Ar}], 7.00 – 6.92 [m, 3H, 4"-H_{Ar} , 2"-H_{Ar}], 5.17 [s, 2H, 5´-H]. **13C-NMR** (75 MHz, DMSO-d6): δ = 168.2 (C, C-7) , 157.9 (C, C_{Ar}-1") , 143.2 (C, C_{Ar}-4´), 138.8 (C, C_{Ar}-2), 137.5 (C, C_{Ar}-1´) , 136.9 (CH, C_{Ar}-4) 133.5 (CH, C_{Ar}-6), 129.4(CH, C_{Ar}-3"), 128.1(CH, C_{Ar}-2´),127.0 (CH, C_{Ar}-3´) , 120.9 (CH, C_{Ar}-4") , 120.5 (CH, C_{Ar}-3) ,

119.0 (C, C_{Ar}-1) , 114.9(CH, C_{Ar}-5), 114.7 (CH, C_{Ar}-2”), 68.0 (CH_2, C-5′) Mp: 175°C; (ESI) m/z: calcd for $C_{20}H_{15}BrNO_5S^-$, 459.9860; found, 459.9878 [M-H]-.

5-bromo-2-(2,4,6-trimethylphenylsulfoamido)benzoic acid (14)

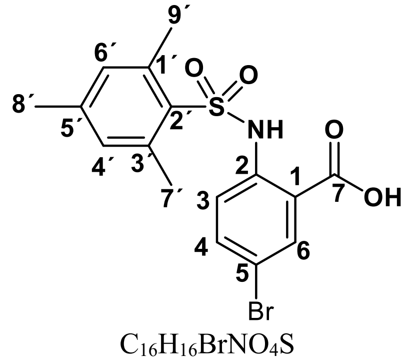

$C_{16}H_{16}BrNO_4S$
M=398.3g/mol

(0.6 g, 78% yield) **^{1}H-NMR** (300 MHz, DMSO-d_6): δ = 11.77 [s,1H, COOH], 9.98 [s, 1H, NH], 7.68 [d, $^4J_{6,4}$ = 2.4 Hz, 1H, 6-H_{Ar}], 7.51[dd, $^3J_{4,3}$ =7.1Hz, $^4J_{4,6}$ =2.4 Hz, 1H 4-H_{Ar}], 7.17 [d, 2H, 4′-H_{Ar} , 6′-H_{Ar}], 7.14 [d, $^3J_{3,4}$= 1H,3-H_{Ar}] , 2.56 [s, 6H, CH_3, 9′-H, 7′-H], 2.21 [s, 3H, CH_3, 8′-H]. **^{13}C-NMR** (300 MHz, DMSO-d_6): δ = 168.8 (C, C-7), 143.3 (C, C_{Ar}-2), 139.5 (C, C_{Ar}-2′), 139.0 and 139.0 (2xC, C_{Ar}-3′, C_{Ar}-1′) 137.3 (CH, C_{Ar}-4), 134.0 (CH, C_{Ar}-6′), 133.0 (CH, C_{Ar}-6), 132.5 and 132.5 (2xCH, C_{Ar}-4′, C_{Ar}-6′) 119.1(CH, C_{Ar}-3), 117.9(C, C_{Ar}-5), 114.3 (C, C_{Ar}-1), 22.5 and 22.5 (2 x CH_3, C-7′, C-9′) 20.7 (CH_3, C-8′) ; mp: 185; MS (ESI): *m/z* calcd for $C_{16}H_{16}BrNO_4S$, 397; found, 398 $[M+H]^+$.

2-(4-acetylphenylsulfoamido)-5-(trifluoromethyl)benzoic acid (15)

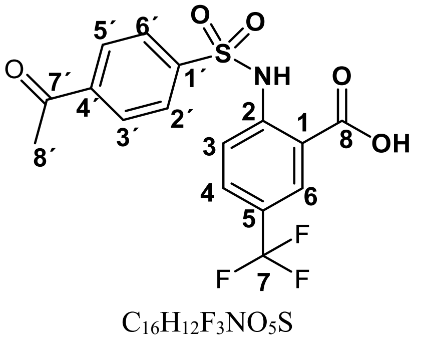

$C_{16}H_{12}F_3NO_5S$
M=387.3g/Mol

(0.4 g, 63% yield) **^{1}H-NMR** (300 MHz, DMSO-d6): δ = 12.28 [s, 1H, COOH]; 12.10 [s, 1H, NH], 8.11 [d, $^4J_{6,4}$= 2.5 Hz, 1H, 6-H_{Ar}], 8.08 [d, $^3J_{3'2'}$ = 7.5 Hz, 2H, 3′-H_{Ar}], 7.86 [dd, $^4J_{4,6}$ = 2.5 Hz, $^3J_{4,3}$ = 7.3Hz, 1H, 4-H_{Ar}], 7.64 [d, $^3J_{4,3}$ = 7.3 Hz, 1H, 3-H_{Ar}] , 7.56 [dd $^3J_{2',3'}$ = 7.5Hz, $^4J_{2',6'}$ =2.3Hz, 2H, 2′-H_{Ar}, 6′-H_{Ar}] 7.22 [dd , $^3J_{3',2'}$ = 7.5Hz , $^4J_{3',5'}$ = 2.1Hz , 2H, 3′-H_{Ar}, 5′-H_{Ar}] 2.50 [s, 3H, CH_3, 8′-H]. **^{13}C-NMR**

(75 MHz, DMSO-d6): δ = 197.9 (C, C-7′), 169.1(C, C-8), 151.8 (C, C_{Ar}-2) 143.5 (C, C_{Ar}-1′), 142.5 (C, C_{Ar}-4′), 140.6 (CH, C_{Ar}-4), 131.4 (CH, C_{Ar}-7), 129.6 (2xCH, C_{Ar}-3′, C_{Ar}-5′), 128.6 (2xCH, C_{Ar}-2′, C_{Ar}-6′), 127.6 (C, C_{Ar}-6), 123.0 (C, C-$_{Ar}$-5), 118.7 (CH, C_{Ar}-3), 27.3 (CH_3, C-8′); mp: 170°C; MS (ESI) *m*/z : calcd. for $C_{16}H_{12}F_3NO_5S$, 387; found, 388 $[M+H]^+$.

2-(2,3-dihydrobenzo[*b*][1,4]dioxine-6-sulfonamido)-5-(trifluoromethyl)benzoic acid (16)

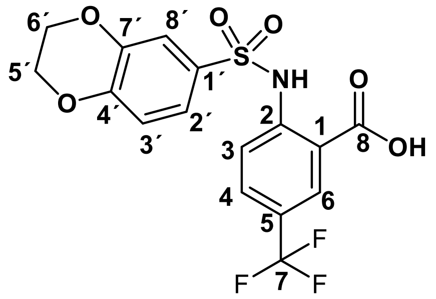

$C_{16}H_{12}F_3NO_6S$
M=403.3g/mol

(0.4 g, 65% yield) **¹H-NMR** (300 MHz, DMSO-d6): δ = 11.48 [s, 1H, COOH], 8.13[s, 1H, NH] , 7.89 [d, $^4J_{6,4}$ = 2.5 Hz , 1H , 6-H_{Ar}] 7.66 [dd, $^3J_{4,3}$ = 7.2 Hz, $^4J_{4,6}$ = 2.5 Hz, 1H, 4-H_{Ar}], 7.23 [d, $^3J_{4,3}$ = 8.1 Hz 1H, 3-H_{Ar}], 7.11 [dd, $^3J_{2',3'}$ = 7.3Hz , $^4J_{2',8'}$= 3.2Hz , 1H, 2′-HAr] 6.95 [d, $^4J_{2',8'}$= 3.2 Hz, 1H , 8′-H_{Ar}] 4.23 – 4.31 [m, 4H, 5′-H, 6′-H]. **¹³C-NMR** (75-MHz, DMSO-d6): δ = 168.9(C, C-8), 148.3(C, C-4′), 143.8 (C, C-2), 143.5 (C, C-7′) , 131.3 (C, C-1′), 130.8 (CH, C-4), 128.6(CH, C-6), 125.7 (C, C-7), 122.1(C, C-5), 120.9(CH, C-2′), 118.3 (CH, C-3), 118.1(CH, C-3′), 116.8 (CH, C-8′) , 64.7(CH_2, C-5′) 64.3 (CH_2, C-6′); mp: 178°C; MS (ESI) *m*/z: calcd. for $C_{16}H_{12}F_3NO_6S$, 403; found, 404 $[M+H]^+$.

5-(trifluoromethyl)-2-(2,4,6-trimethylphenylsulfoamido)benzoic acid (17)

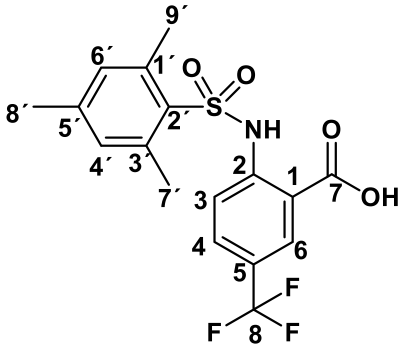

$C_{17}H_{16}F_3NO_4S$
M=387.4g/mol

(0.38 g, 62% yield) ^{1}**H-NMR** (300 MHz, DMSO-d6): δ = 12.28 [s, 1H, COOH], 11.60 [s, 1H, NH], 8.15 [d, $^{4}J_{6,4}$ = 2.1 Hz, 1H, 6-H_{Ar}] 7.92 [dd, $^{3}J_{4,3}$ =7.9 Hz, $^{4}J_{4,6}$= 2.1 Hz, 1H,4-H_{Ar}] 7.87 [d, $^{4}J_{6',4'}$= 1.9 Hz, 2H, 4´-H_{Ar} , 6´-H_{Ar}], 7.48 [d, $^{3}J_{3,4}$ = 7.9 Hz, 1H, 3-H_{Ar}] , 2.60 [s, 6H, CH_3 , 9´-H, 7´-H], 2.23 [s, 3H, CH_3, 8´-H]. ^{13}**C-NMR** (75 MHz, DMSO-d6): δ = 169.3 (C, C-7), 154.2 (C, C-2), 143.6 (C, C-2´) , 139.1 and 139.1 (2xC, C-1´, C-3´) 132.9 (C, C-5´), 132.5 (CH, C-4), 131.5 and 131.5 (2xCH, C-4´, C-6´) , 130.1(CH, C-6), 128.7 (C, C-8), 122.5 (C, C-5), 117.0 (CH, C-3), 109.0 (C, C-1) ,22.4 and 22.4 (2xCH_3,C-7´, C-9´), 20.8 (CH_3, C-8´) ; mp:184°C; MS (ESI) *m*/z: calcd. for $C_{17}H_{16}F_3NO_4S$, 387; found, 388 $[M+H]^+$.
